# S100A3 a partner protein regulating the stability/activity of RARα and PML-RARα in cellular models of breast/lung cancer and acute myeloid leukemia

**DOI:** 10.1038/s41388-018-0599-z

**Published:** 2018-12-07

**Authors:** Maurizio Gianni, Mineko Terao, Mami Kurosaki, Gabriela Paroni, Laura Brunelli, Roberta Pastorelli, Adriana Zanetti, Monica Lupi, Andrea Acquavita, Marco Bolis, Maddalena Fratelli, Cecile Rochette-Egly, Enrico Garattini

**Affiliations:** 1 Laboratory of Molecular Biology, Istituto di Ricerche Farmacologiche Mario Negri IRCCS, via La Masa 19, 20156 Milano, Italy; 2 Laboratory of Mass Spectrometry, Istituto di Ricerche Farmacologiche Mario Negri IRCCS, via La Masa 19, 20156 Milano, Italy; 3 Laboratory of Cancer Pharmacology, Istituto di Ricerche Farmacologiche Mario Negri IRCCS, via La Masa 19, 20156 Milano, Italy; 4 Department of Functional Genomics and Cancer, IGBMC (Institut de Génétique et de Biologie Moléculaire et Cellulaire), INSERM, U964; CNRS, UMR7104, Université de Strasbourg, 1 rue Laurent Fries, BP 10142, 67404 Illkirch Cedex, France

**Keywords:** Proteomics, Acute myeloid leukaemia

## Abstract

All trans-retinoic acid (ATRA) is used in the treatment of acute promyelocytic leukemia (APL) and it is a promising agent also in solid tumors. The pharmacological activity of ATRA is mediated by the ligand-activated RAR and RXR transcription factors. In the present study, we define the basal and ATRA dependent RARα interactome in a RARα-overexpressing breast cancer cellular model, identifying 28 nuclear proteins. We focus our attention on the S100A3 calcium-binding protein, which interacts with RARα constitutively. In ATRA-sensitive breast cancer cells, S100A3 binds to RARα in basal conditions and binding is reduced by the retinoid. The interaction of S100A3 with RARα is direct and in lung cancer, APL and acute-myeloid-leukemia (AML) cells. In APL, S100A3 interacts not only with RARα, but also with PML-RARα. The interaction surface maps to the RARα ligand-binding domain, where the *I396* residue plays a crucial role. Binding of S100A3 to RARα/PML-RARα controls the constitutive and ATRA-dependent degradation of these receptors. S100A3 knockdown decreases the amounts of RARα in breast- and lung cancer cells, inducing resistance to ATRA-dependent anti-proliferative/differentiating effects. Conversely, S100A3 knockdown in PML-RARα^+^ APL and PML-RARα^−^ AML cells reduces the amounts of RARα/PML-RARα and increases basal and ATRA-induced differentiation. In this cellular context, opposite effects on RARα/PML-RARα levels and ATRA-induced differentiation are observed upon S100A3 overexpression. Our results provide new insights into the molecular mechanisms controlling RARα activity and have practical implications, as S100A3 represents a novel target for rational drug combinations aimed at potentiating the activity of ATRA.

## Introduction

All-trans retinoic acid (ATRA) is used in the treatment of acute promyelocytic leukemia (APL) [1, 2], which is characterized by a chromosomal translocation involving the retinoid-receptor, RARα, resulting in the production of the PML-RARα oncogene [2–4]. ATRA is a promising agent in the treatment/chemoprevention of other neoplastic diseases, including breast cancer [3].

ATRA activity is mediated by the ligand-activated transcription factors, RARα, RARβ, RARγ, RXRα, RXRβ, and RXRγ [4, 5]. These proteins consist of six regions A, B, C, D, E, and F from the N- to the C-terminus. The C-region is involved in DNA-binding, while the E-region contains the ligand-binding pocket [6–9]. Transcriptionally active retinoid-receptors consist of RAR/RXR heterodimers, in which RARs acts as the ligand-binding moiety. Unliganded RAR/RXR heterodimers bind to RAREs (retinoic-acid-responsive-elements) located in the regulatory regions of retinoid target-genes [6, 10]. Ligand-free RAR/RXR heterodimers are part of multi-protein complexes whose composition is modified upon ligand-binding. Specific RARs and RXRs interact with different proteins which modulate their transcriptional activity, phosphorylation/dephosphorylation [11], degradation [12] and subcellular localization [13].

Here, we use a proteomic approach to identify novel proteins interacting with RARα. Among the proteins showing a strong interaction with RARα, we focus on the calcium-binding protein, S100A3. The interaction of S100A3 with RARα is observed in breast cancer and lung cancer, acute-myeloid-leukemia (AML) as well as acute promyelocytic leukemia (APL) cells, in which S100A3 binds also to PML-RARα. S100A3 binding controls constitutive and ATRA-dependent degradation of RARα and PML-RARα. This interaction has opposite consequences on the anti-proliferative and differentiating activity of ATRA in breast cancer and lung cancer relative to AML and APL cells.

## Results

### Identification of RARα interacting proteins

RARα is the major determinant of ATRA anti-tumor activity in breast cancer [3, 5]. To identify novel RARα interacting proteins, we stably transfected a FLAG-tagged RARα (*FLAG-RARα*) plasmid and the corresponding void vector in ATRA-resistant *MDA-MB-453* breast cancer cells [3, 14], generating *RA-453* and *FL-453* cells, respectively. In basal conditions, *RA-453* express higher levels of RARα than parental *MDA-MB-453* and *FL-453* cells (Supplementary Fig. 1A). Unlike *MDA-MB-453* and *FL-453*, *RA-453* cells are responsive to the transcriptional and growth-inhibitory effects of ATRA. In fact, *RA-453* cells transfected with a retinoid-dependent luciferase reporter (*DR5-RARELuc*) show stimulation of luciferase activity by ATRA. In addition, *RA-453* growth is reduced by ATRA in a dose-dependent manner (Supplementary Fig. 1B).

To screen for RARα-binding proteins in *RA-453* and *FL-453* cells, we used a quantitative proteomic approach [15, 16] (Supplementary Fig. 2). Nuclear fractions enriched for DNA-binding (*NABP, nucleic acid binding proteins*) and histonic (*INP, insoluble nuclear proteins*) proteins were extracted from *RA-453* and *FL-453* cells exposed to vehicle or ATRA. Each nuclear fraction was immunoprecipitated with anti-FLAG antibodies and subjected to proteomic analysis. Twenty-eight of the proteins identified are present only in the *RA-453* immunoprecipitates (Supplementary Table 1 and Supplementary Table 2). Ten proteins bind to unliganded RARα and binding is increased by at least 1.5-fold following treatment with ATRA (Supplementary Table 1). With the exception of CEP83 [17] and RL1D1 [18], all these interactors are histone proteins. Interestingly, CEP83 and RL1D1 are contained in the *INP* fraction. Thus, RARα-binding of these proteins may be indirect and mediated by one of the identified histones. The H2AW core-histone protein shows maximal ATRA-dependent stimulation of RARα-binding.

Seventeen proteins, none of which is a histone, are identifiable in the *NABP* and *INP* fractions of vehicle-treated *RA-453* cells (Supplementary Table 2). RARα-binding of all these proteins is reduced by ATRA.

### S100A3, FABP5, and HSPB1 bind to unliganded RARα and the interaction is diminished by ATRA

**Fig. 1:**
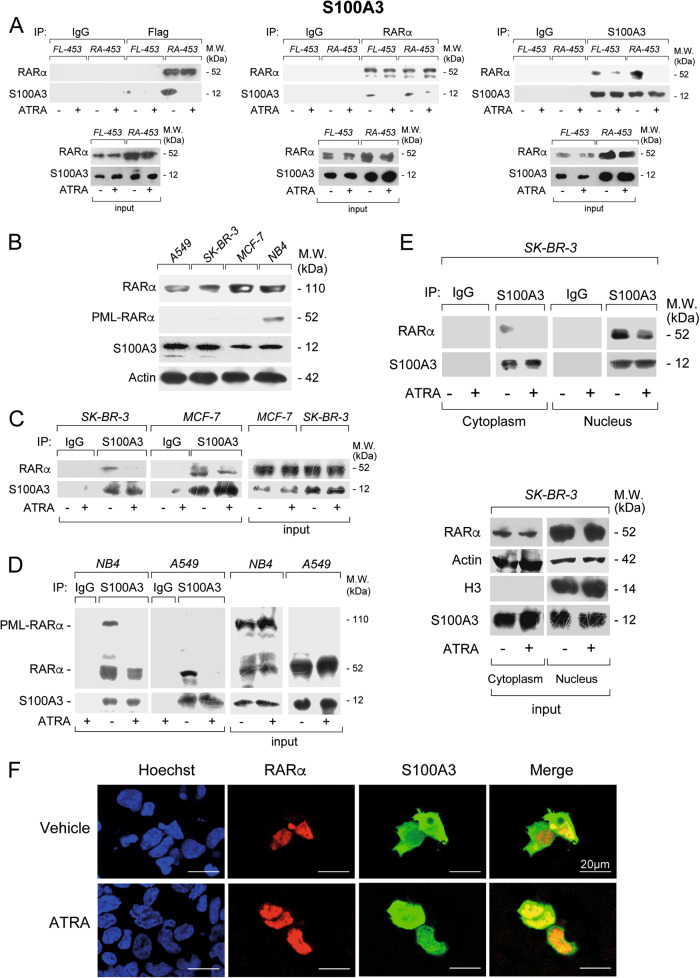
Interactions between S100A3 and RARα in breast cancer *FL-453*, *RA-453*, *SK-BR-3*, and *MCF7* cells, lung cancer *A549* cells as well as APL-derived *NB4* cells. **a** *FL-453* and *RA-453* cells were treated with vehicle (DMSO) or ATRA (1 µM) for 1 h. At the end of the treatment, total cell extracts were immunoprecipitated with anti-FLAG mouse monoclonal antibodies (left), anti-RARα mouse monoclonal antibody (middle), and anti-S100A3 mouse monoclonal antibodies (right) or the corresponding non-specific immuno-globulins G (IgG) as negative controls. Following normalization for the content of RARα or S100A3 in the input, the various immunoprecipitates were subjected to western blot analysis with an anti-RARα rabbit polyclonal antibody or the anti-S100A3 antibody, as indicated. M.W. = molecular weights of the indicated proteins. Input = western blot analysis of the cell extracts before the indicated immunoprecipitation step. Each immunoprecipitation is representative of at least two independent experiments providing the same type of results. **b** Extracts from logarithmically growing breast cancer *SK-BR-3* and *MCF7* cells, lung cancer *A549* cells as well as APL-derived *NB4* cells were subjected to western blot analysis with antibodies targeting RARα and PML-RARα, S100A3, and β-actin. The molecular weights of the indicated proteins are shown on the right. *SK-BR-3* and *MCF7* (**c**) as well as *NB4* and *A549* (**d**) cells were treated with vehicle (DMSO) or ATRA (1 µM) for 1 h. At the end of the treatment, total cell extracts were immunoprecipitated with anti-S100A3 mouse monoclonal antibodies (IP: S100A3). The negative control for the immunoprecipitations is represented by the extracts challenged with non-specific immuno-globulins G (IP: IgG), as indicated. Following normalization for the content of S100A3 in the input, the immunoprecipitates were subjected to western blot analysis with anti-RARα and anti-S100A3 antibodies, as indicated. Input = western blot analyses of the cell extracts before the immunoprecipitation step. M.W. = molecular weights of the indicated proteins. **e** *SK-BR-3* cells were treated with vehicle (DMSO) or ATRA (1 µM) for 1 h. At the end of the treatment, the nuclear (Nucleus) and the cytoplasmic (Cytoplasm) fractions of the cells were separated by centrifugation and subjected to immunoprecipitation with the anti-S100A3 antibody or the control IgG. As in (**c**), the immunoprecipitates were subjected to western blot analysis with anti-RARα and anti-S100A3 antibodies. Input = western blot analyses of the cell extracts before the immunoprecipitation step. The input data obtained with the nuclear marker, Histone H3 (H3), demonstrate efficient separation of the nuclear from the cytoplasmic fractions. Each immunoprecipitation is representative of at least two independent experiments providing the same type of results. **f** *MDA-MB-453* cells were co-transfected with RARα and S100A3 expression plasmids. Twenty-four hours following transfection, cells were challenged with primary anti-RARα rabbit polyclonal antibodies mouse and anti-S100A3 monoclonal antibodies. Subsequently the cell slides were labeled with red-fluorescent anti-rabbit-Ig and green fluorescent anti-mouse-Ig secondary antibodies. Cell nuclei are shown in blue following staining with the Hoechst dye. Merging of the red- and green-fluorescence images is shown in the rightmost panels

We focused on the three RARα interactors, S100A3, FABP5, and HSPB1. S100A3 is a calcium-binding protein involved in transcription [19–21]. FABP5 protein binds and delivers ATRA to the PPARβ/δ nuclear-receptor [22–24]. HSPB1/HSP27 is a heat-shock protein whose expression is modulated by ATRA [25–27]. Detectable levels of FABP5, HSPB1/HSP27 (Supplementary Fig. 3A and Supplementary Fig. 3B), and S100A3 (Fig. 1a) are observed in vehicle and ATRA-treated *FL-453* as well as *RA-453* cells. ATRA treatment does not alter the basal expression of the three proteins.

We validated the observed interactions of S100A3, FABP5, and HSPB1/HSP27 with RARα using co-immunoprecipitation studies (Fig. 1a and Supplementary Fig. 3A, B). Anti-FLAG antibodies immunoprecipitate RARα only in *RA-453* cells and the amount of immunoprecipitated RARα is similar in vehicle- and ATRA-treated *RA-453* cells. In *RA-453* cells, S100A3 (Fig. 1a), FABP5 (Supplementary Fig. 3A), and HSPB1/HSP27 (Supplementary Fig. 3B) co-immunoprecipate with RARα and the levels of the three co-immunoprecipitated proteins are higher with vehicle than ATRA. This confirms the proteomic results and indicates that S100A3, FABP5, and HSPB1/HSP27 interact predominantly with unliganded RARα. The interaction of S100A3, FABP5, and HSPB1/HSP27 with RARα in *RA-453* cells is also observed if the anti-FLAG immunoprecipitating antibodies are substituted by anti-RARα antibodies. Noticeably, anti-RARα antibodies immunoprecipitate not only FLAG-tagged RARα, but also endogenous RARα, which is expressed in both *RA-453* and *FL-453* cells, albeit at low levels. Further evidence supporting specific interactions between RARα and these proteins comes from the co-immunoprecipitation studies with anti-FABP5, anti-HSPB1 or anti-S100A3 antibodies.

### RARα/S100A3 interaction is a general phenomenon and it occurs in the nucleus

We concentrated on S100A3, as the relevance of this protein for the biological activity of ATRA is unknown. To evaluate whether S100A3/RARα interaction is a general phenomenon, we performed co-immunoprecipitation studies in ATRA-sensitive tumor cell lines of different origin. We considered breast cancer *SK-BR-3* and *MCF-7* cells [3], lung cancer *A-549* cells [28] as well as APL-derived and PML-RARα^+^ *NB4* blasts [29, 30] which synthesize measurable levels of S100A3 and RARα (Fig. 1b). RARα and S100A3 are detectable in the anti-S100A3 immunoprecipitates of vehicle-treated *SK-BR-3*, *MCF-7*, *A-549*, and *NB4* cells (Fig. 1c, d). ATRA reduces the amounts of co-immunoprecipitated RARα, regardless of the cell line considered. In *NB4* cells, S100A3 interacts also with PML-RAR and the interaction is reduced by ATRA (Fig. 1d).

We evaluated the subcellular distribution of the RARα/S100A3 interaction in *SK-BR-3* cells, using quantitative immunoprecipitation, following separation of the nuclear and cytoplasmic fractions (Fig. 1e). As expected, RARα localizes predominantly to the nucleus [31–33], while S100A3 is evenly distributed in the cytosol and the nucleus (Fig. 1e-input). In basal conditions, the anti-S100A3 antibody co-immunoprecipitates significant amounts of RARα only from the nucleus and RARα nuclear protein levels are reduced by ATRA. Immunofluorescence experiments performed in *MDA-MB-453* cells transiently transfected with RARα and S100A3 support these results (Fig. 1f), demonstrating punctuate co-localization of RARα and S100A3 only inside the nucleus.

### S100A3 interacts with RARα, PML-RARα, and RARγ

**Fig. 2:**
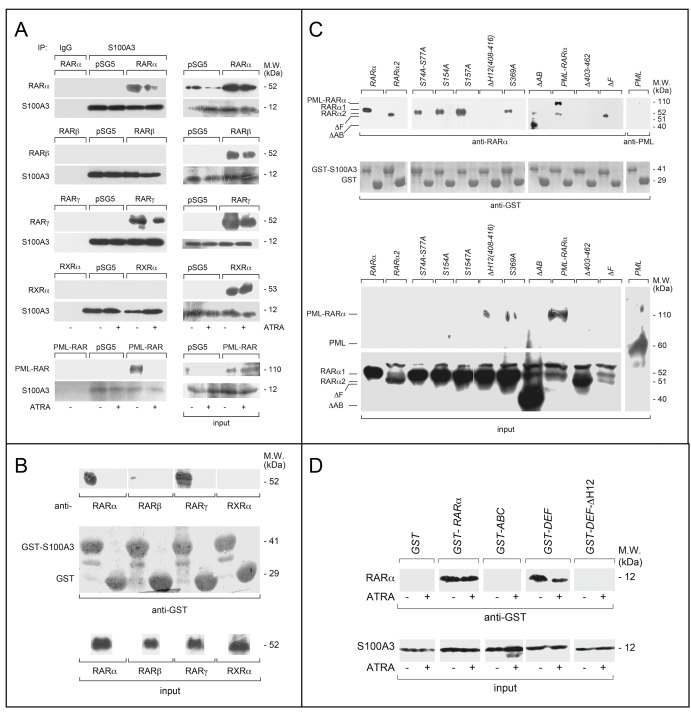
Specificity and structural determinants of RARα binding to S100A3. **a** *COS-7* cells were co-transfected with equal amounts of RARα, PML-RARα, RARβ, RARγ or RXRα, and S100A3 expression plasmids, as indicated. The negative control for the experiments is represented by cells co-transfected with the void expression plasmid (*pSG5*). Twenty-four hours following transfection, cells were treated with vehicle (DMSO) or ATRA (1 µM) for 1 h. At the end of the treatment, total cell extracts were immunoprecipitated with anti-S100A3 mouse monoclonal antibodies (IP: S100A3). A further negative control for the immunoprecipitations is represented by the extracts of *COS-7* cells co-transfected with *pSG5* and the S100A3 expression plasmid which were challenged with non-specific immuno-globulins G (IP: IgG). Following normalization for the content of S100A3 in the input, the various immunoprecipitates were subjected to western blot analysis with anti-RARα, anti-RARβ, anti-RARγ, or anti-RXRα antibodies. All the blots were subsequently re-challenged with anti-S100A3 antibodies, as indicated. Input = western blot analysis of the cell extracts before the indicated immunoprecipitation step. Each immunoprecipitation is representative of at least two independent experiments providing the same type of results. **b**, **c** GST pull-down: the GST-tagged recombinant protein, GST-S100A3, and the GST negative control were used. The two recombinant proteins conjugated to Glutathione-Sepharose beads were incubated with extracts of *COS-7* cells transfected with the *pSG5* expression plasmids containing wild-type RARα, RARβ, RARγ, RXRα, RARα2, PML-RARα and the indicated RARα deletion-mutants and point-mutants. GST pull-down precipitates were blotted on nitro-cellulose filters, incubated with an anti-RARα, anti-RARβ, anti-RARγ, anti-RXRα antibodies (**b**) or anti-RARα antibodies only (**c**). Subsequently the filters were re-blotted with an anti-GST antibody, as indicated. Input: cell extracts (15 μg of protein) representing 10% of the total amount of protein were subjected to western blot analysis with the above anti-RARα antibody. **d** Far-western: *COS-7* cells were transfected with the same S100A3 expression plasmid as in (**a**). Cell extracts were precipitated with sepharose beads conjugated with an anti-S100A3 monoclonal antibody. The immunoprecipitates were subjected to far-western analysis using the following GST-tagged RARα recombinant proteins: *GST-RARα* = full-length RARα; *GST-ABC* = RARα ABC regions; *GST-DEF* = RARα DEF regions; *GST-DEFΔH12* = RARα1 DEF regions lacking the H12-helix. The blots were developed with an anti-GST antibody. Input: cell extracts (15 μg of protein) representing 10% of the total amount of protein used for the immunoprecipitations were subjected to western blot analysis with an anti-S100A3 antibody. Each line shows cropped lanes of the same gel, hence the results can be compared across the lanes, as they were obtained with the same exposure time. M.W. = molecular weights of the indicated proteins

To validate the observation that S100A3 interacts with RARα/PML-RARα and to evaluate whether the calcium-binding protein recognizes other members of the RAR/RXR family, we used the *COS-7* cellular model [31]. We performed co-immunoprecipitation experiments following overexpression of S100A3 with RARα, PML-RARα, RARβ, RARγ, or RXRα (Fig. 2a). RARα and PML-RARα are not the only S100A3-interacting retinoid-receptors, as anti-S100A3 antibodies co-immunoprecipitate also RARγ. As expected, binding of RARα, PML-RARα, and RARγ to S100A3 is reduced by ATRA. To confirm these results, we performed GST pull-down assays [32] in *COS-7* cells over-expressing RARα, RARβ, RARγ, and RXRα. In basal conditions, only RARα and RARγ are specifically pulled down by GST-tagged S100A3 (*GST-S100A3*, Fig. 2b).

### Definition of the RARα structural determinants of the interaction with S100A3

To get insights into the structural determinants of the RARα/S100A3 interaction, we performed GST pull-down assays using deletion-/phosphorylation-mutants of the retinoid-receptor. We over-expressed the following RARα deletion-mutants in *COS-7* cells: *ΔAB*, deleted for the A/B-regions; *ΔH12(408–416)*, deleted for the H12-helix in the ligand-binding E-region; *Δ403–462*, deleted for a portion of the E- and the entire F-region; *ΔF*, deleted for the F-region [9, 33]; *ΔC*, deleted for the DNA-binding C-region; *ΔD*, deleted for the D-hinge-region (Supplementary Fig. 4). In addition, we over-expressed PML-RARα and the RARα2 splicing-variant [32] and used *PML* as an internal control. Only *ΔH12(408–416)* and *Δ403–462* lose the ability to interact with S100A3 (Fig. 2c). Deletion of the C- and D-regions causes a major reduction of the S100A3-interaction, although it does not abrogate binding (Supplementary Fig. 5). This last observation must be taken with caution as the absence of the C- or D-regions has major effects on the tridimensional structure of the RAR protein. Thus, the GST pull-down experiments confirm that PML-RARα interacts with S100A3. This interaction is due to the RARα moiety, as PML is not recognized by GST-S100A3.

**Fig. 3:**
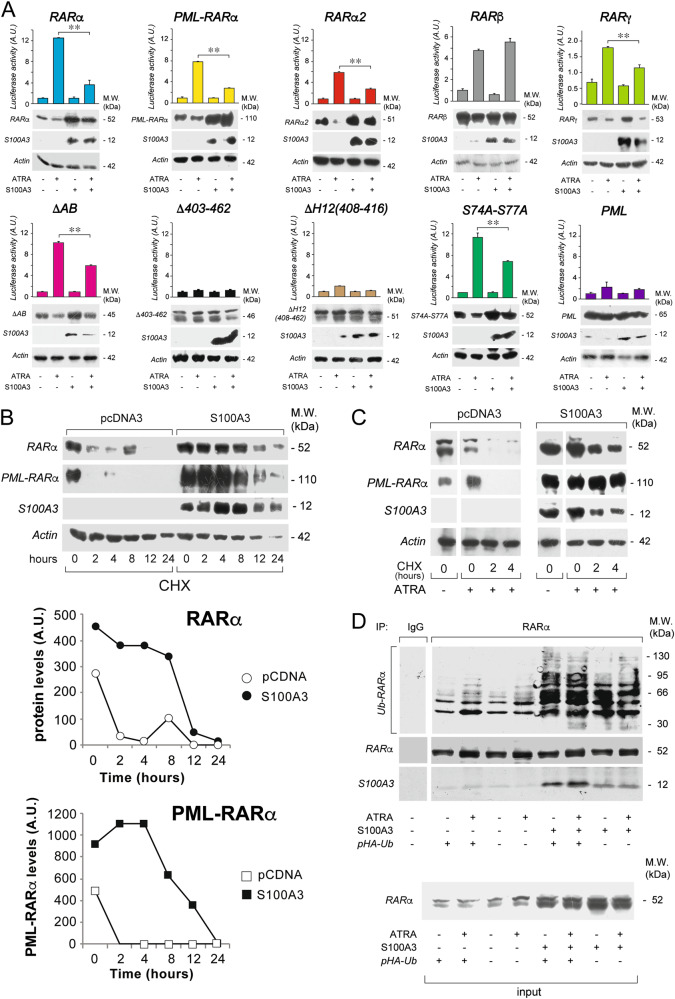
RARα structural determinants of the interaction with S100A3 and effects of S100A3 on the stability and ubiquitinylation of the retinoid-receptor and the PML-RARα fusion product. **a** *COS-7* cells were transiently co-transfected with an expression plasmid for the S100A3 cDNA (*S100A3*) or the corresponding void vector (*pcDNA3*) and wild-type RARα, PML-RARα, RARα2, RARβ, RARγ, the indicated RARα deletion-mutants and point-mutants along with a luciferase reporter construct controlled by a retinoid responsive element (*β2RARE-Luc*). PML was used as an internal negative control of the experiment. Twenty-four hours following transfection, cells were treated with vehicle (DMSO) or ATRA (1 µM) for a further 24 h. Cell extracts were subjected to western blot analysis with anti-RARα, anti-RARβ, anti-RARγ, anti-PML (upper panels), anti-S100A3 (middle panels), and anti-actin (lower panels) antibodies. The same cell extracts were used for the measurement of luciferase activity, as illustrated by the bar graphs above the western blots. Each value is the mean ± SD of three replicate cultures. **Statistically significant comparison (*p* < 0.01, Student’s *t*-test). **b** *COS-7* cells were transiently co-transfected with *S100A3* or *pcDNA3* and wild-type RARα or PML-RARα. Upper: twenty-four hours following transfection, cells were split and treated with cycloheximide (CHX, 10 µg/ml) for the indicated amounts of time. Lower: the graphs indicate the results obtained following densitometric analysis of the western blot signals obtained for RARα and PML-RARα. Densitometric analysis was performed with the Progenesis software (Nonlinear Dynamics Co.). **c** *COS-7* cells were transfected as in (**b**). Twenty-four hours following transfection cells were split and treated with ATRA (1 µM) for another 24 h. Subsequently cells were exposed to CHX (10 µg/ml) for the indicated amounts of time. Cell extracts were subjected to western blot analysis with anti-RARα, anti-S100A3, and anti-actin antibodies, as indicated. Each line shows cropped lanes of the same gel, hence the results can be compared across the lanes, as they were obtained with the same exposure time. **d** *COS-7* cells were transiently co-transfected with *S100A3* or *pcDNA3* and wild-type RARα in the presence/absence of an HA-tagged ubiquitin expression vector (*pHA-Ub*). Twenty-four hours following transfection, cells were treated with vehicle (DMSO) or ATRA (1 µM) for 1 h. Cell extracts were immunoprecipitated with an anti-RARα mouse monoclonal antibody (IP: RARα) or with non-specific immuno-globulins G (IP: IgG) and subjected to western blot analysis with an anti-ubiquitin (upper) and anti-RARα rabbit polyclonal (lower) antibodies. *Ub-RARα* = poly-ubiquitinylated RARα. M.W. = molecular weights of the indicated proteins

RARα is phosphorylated at different residues in basal conditions or following exposure to ATRA [11]. Specific phosphorylation sites control the levels and functional activity of the retinoid-receptor. To evaluate whether some of the known phosphorylation sites play a role in the S100A3/RARα interaction, we performed GST pull-down studies with available Ser/Ala phosphorylation-mutants. We used the following phosphorylation-mutants: *S74A-S77A*, inactivating two p38 and CDK dependent phosphorylation sites of the B-domain [34, 35]; *S154A* and *S157A*, inactivating the PKC-dependent phosphorylation sites of the C-domain [36]; *S369A*, inactivating a PKA/p38-dependent phosphorylation site in the E-domain [37] (Supplementary Fig. 4). None of the four phosphorylation mutant exerts a significant effect on the interaction with S100A3 (Fig. 2c). As ATRA activates the phosphorylation of all these mutated Ser-residues [11], the amino-acids are also unlikely to be involved in the diminution of RARα/S100A3 interaction caused by the retinoid (see also Fig. 3a, *S74A-S77A*).

To establish whether the interaction between S100A3 and RARα is direct, far-western experiments [32] were performed in *COS-7* cells over-expressing S100A3 (Fig. 2d). The results demonstrate that GST-RARα interacts with S100A3. The interaction is reproduced with the GST-RARα derivative consisting of the DEF regions (*GST-DEF*) [32], but not with similar proteins consisting of the *ABC*-regions (*GST-ABC*) or the DEF regions lacking the H12-helix (*GST-DEFΔH12*). This demonstrates that S100A3 interacts with RARα in a direct manner and confirms the importance of the H12-helix in the interaction.

### S100A3 controls the transcriptional activity and the degradation of RARα and PML-RARα

To evaluate whether the interaction with S100A3 exerts any effect on the transcriptional activity and the levels of the two retinoid-receptors, we co-transfected *COS-7* cells with S100A3 and RARα or PML-RARα in the presence of a retinoid-dependent luciferase reporter. S100A3 reduces ATRA-dependent stimulation of RARα and PML-RARα, as assessed by the luciferase reporter (Fig. 3a). In vehicle-treated *COS-7* cells, *S100A3* upregulates both RARα and PML-RARα proteins. In the absence of S100A3, ATRA reduces the amounts of the two receptor proteins [12, 32], a phenomenon which is not observed in S100A3 over-expressing cells. The effects exerted by S100A3 on the levels of RARα and PML-RARα proteins are translational/posttranslational, as they are not accompanied by alterations in the amounts of the corresponding mRNAs (data not shown). The data suggest that S100A3 increases the levels of a transcriptionally-inactive form of RARα and PML-RARα. The specificity of the results is confirmed with the use of PML as a negative control.

To support the idea that the results obtained are due to the S100A3-interaction, we performed similar experiments with RARα2, RARβ, RARγ, and RARα deletion- or point-mutants maintaining (*ΔAB* and *S74A-S77A*) or losing (*ΔH12(408–416)* and *Δ403–462*) the ability to bind S100A3 (Fig. 3a). S100A3 increases the protein levels and inhibits the ATRA-dependent luciferase activity of RARα2, RARγ, *ΔAB,* and *S74A-S77A*. In contrast, the amounts of RARβ, *ΔH12(408–416),* and *Δ403–462* proteins are not modified by S100A3 overexpression. In the case of RARβ protein, S100A3 has also no influence on ATRA-dependent luciferase activity.

To determine the effects of S100A3 on the stability of the two proteins, *COS-7* cells were transfected with S100A3 and RARα or PML-RARα before exposure to cycloheximide for different amounts of time in the absence (Fig. 3b) and presence of ATRA (Fig. 3c). In basal conditions, S100A3 increases the stability of RARα and PML-RARα (Fig. 3b, lower graphs). By the same token, S100A3 reduces ATRA-dependent degradation of the two retinoid-receptors, a phenomenon associated with ligand-dependent transcriptional activation [29]. As the proteasome is involved in the degradation of the two receptors [38], we evaluated the effects of S100A3 on the ubiquitinylation of RARα. RARα, S100A3, and HA-tagged ubiquitin (*pUb-HA*) were over-expressed in *COS-7* cells exposed to vehicle or ATRA. Cell extracts were immunoprecipitated with anti-RARα antibodies and blotted with antibodies recognizing poly-ubiquitinylated proteins (Fig. 3d). Regardless of ATRA treatment, which stimulates the process [39], S100A3 increases RARα polyubiquitinylation and the effect is magnified by *pUb-HA*. This suggests that S100A3/RARα interaction inhibits constitutive and ATRA-dependent degradation of the retinoid-receptor by the proteasome.

### The I396 residue of RARα is critical for the interaction with S100A3

Constitutive binding to RARα/PML-RARα, reduction of the binding by ATRA and inhibition of RARα/PML-RARα transcriptional activity suggest that S100A3 is a potential nuclear-receptor co-repressor. In addition, S100A3 contains a LKELLQKEL sequence (Supplementary Fig. 6), which is similar to the core alpha-helical-box (LXXI/HIXXXIL) of co-repressors [40].

**Fig. 4:**
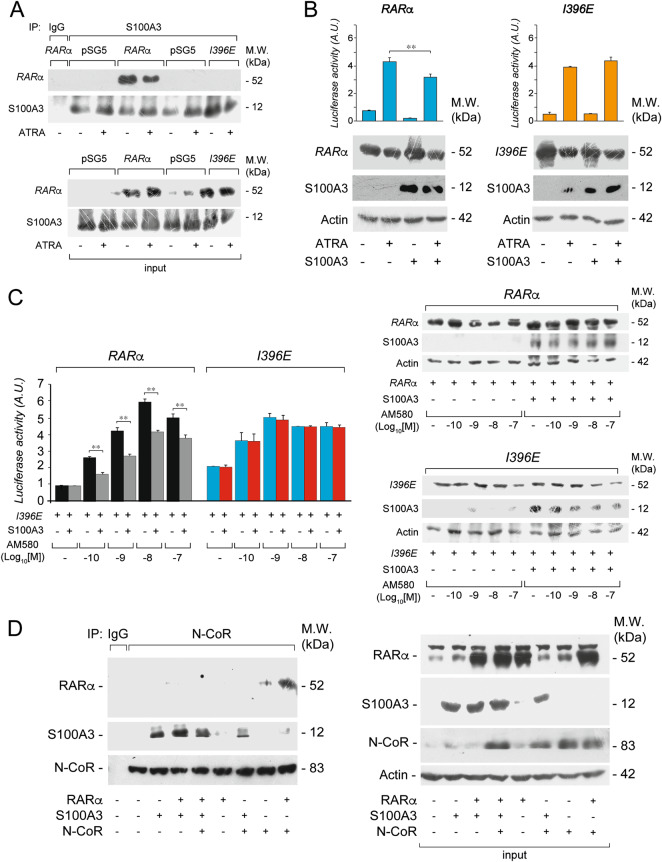
Interaction studies between S100A3, the RARα I396E mutant and the co-repressor N-COR. **a** *COS-7* cells were co-transfected with equal amounts of wild-type RARα, *I396E*, and S100A3 expression plasmids, as indicated. The negative control for the experiments is represented by cells co-transfected with the void expression plasmid (*pSG5*). Twenty-four hours following transfection, cells were treated with vehicle (DMSO) or ATRA (1 µM) for 1 h. At the end of the treatment, total cell extracts were immunoprecipitated with anti-S100A3 mouse monoclonal antibodies (IP: S100A3). A further negative control for the immunoprecipitations is represented by the extracts of *COS-7* cells co-transfected with *pSG5* and the S100A3 expression plasmid which were challenged with non-specific immuno-globulins G (IP: IgG). Following normalization for the content of S100A3 in the input, the various immunoprecipitates were subjected to western blot analysis with anti-RARα antibodies. All the blots were subsequently re-challenged with anti-S100A3 antibodies, as indicated. Input = western blot analysis of the cell extracts before the indicated immunoprecipitation step. M.W. = molecular weights of the indicated proteins. Each immunoprecipitation is representative of at least two independent experiments providing the same type of results. **b** *COS-7* cells were transiently co-transfected with an expression plasmid for the S100A3 cDNA (*S100A3*) or the corresponding void vector (*pcDNA3*), wild-type RARα and the RARα *I396E* mutant (*I396E*) along with a luciferase reporter construct controlled by a retinoid responsive element (*β2RARE-Luc*). Twenty-four hours following transfection, cells were treated with vehicle (DMSO) or ATRA (1 µM) for a further 24 h. Cell extracts were subjected to western blot analysis with anti-RARα (upper panels), anti-S100A3 (middle panels) and anti-actin (lower panels) antibodies. The same cell extracts were used for the measurement of luciferase activity, as illustrated by the bar graphs above the western blots. M.W. = molecular weights of the indicated proteins. **c** *COS-7* cells were transiently transfected with the indicated expression plasmids as in (**b**). Twenty-four hours following transfection, cells were exposed to the indicated concentrations of the AM580 RARα agonist. Left: the bar graph illustrate the levels of luciferase activity. Each value is the mean ± SD of three replicate cultures. **Statistically significant comparison (*p* < 0.01, Student’s *t*-test). Right: the same extracts used for the determination of luciferase activity were subjected to western blot analysis with anti-RARα (upper panels), anti-S100A3 (middle panels) and anti-actin (lower panels) antibodies. **d** *COS-7* cells were transiently co-transfected with the indicated combinations of *S100A3*, RARα, and a N-CoR fragment (aa. 1629–2453) containing the RARα-binding domains (NRI and NRII). Following normalization for the content of N-COR in the input, cell extracts were immunoprecipitated with an anti-N-COR antibody (IP: N-COR) or with non-specific immuno-globulins G (IP: IgG) and subjected to western blot analysis with anti-RARα, anti-S100A3, and anti-N-COR antibodies. Input = western blot analysis of the cell extracts before the indicated immunoprecipitation step. M.W. = molecular weights of the indicated proteins

The I396 residue is located in proximity to the RARα region involved in S100A3 binding (Supplementary Fig. 4) and the RARα I396E mutant (*I396E*) does not bind co-repressors [41]. Thus, we evaluated the capacity of *I396E* to bind S100A3, in the *COS-7* model. Unlike RARα, *I396E* is not co-immunoprecipitated by anti-S100A3 antibodies (Fig. 4a). Consistent with the inability of S100A3 to interact with *I396E*, the calcium-binding protein has no effect on ATRA-dependent transcriptional activity or the steady-state levels of the RARα-mutant (Fig. 4b). As ATRA is a pan-RAR agonist, we supported the selectivity of these effects with the RARα agonist, AM580 [4]. We compared the dose-dependent effects of AM580 on RARα and *I396E* transcriptional activity (Fig. 4c, left). As for RARα, S100A3 reduces the luciferase activity of the retinoid-dependent *RARβ2-TKLuc* reporter, at all AM580 concentrations. In contrast, S100A3 does not affect the AM580-dependent transcriptional activity of *I396E*. Transcriptional inhibition by AM580 is accompanied by RARα-protein stabilization, while a similar effect is not observed with *I396E* (Fig. 4c, right).

We also evaluated whether S100A3 competes with the N-CoR co-repressor for RARα binding and we performed co-immunoprecipitation studies in *COS-7* cells over-expressing combinations of the three proteins (Fig. 4d). For these studies, we used an N-CoR fragment (aa. 1629–2453) containing the RARα-binding domains [42]. Cell extracts were immunoprecipitated with anti-N-CoR antibodies and subjected to western blot analysis for RARα or S100A3. N-CoR antibodies co-immunoprecipitates RARα in *N-COR* and more so in *N-COR* + *RARα* transfected cells. Overexpression of S100A3 (*N-COR* + *S100A3* and *N-COR* + *RARα* + *S100A3* cells) competes for the binding of N-COR to RARα. This is consistent with S100A3 and N-COR binding to the same H12 region of RARα.

### Effects of S100A3 on the cellular responses to ATRA in breast cancer cells

**Fig. 5:**
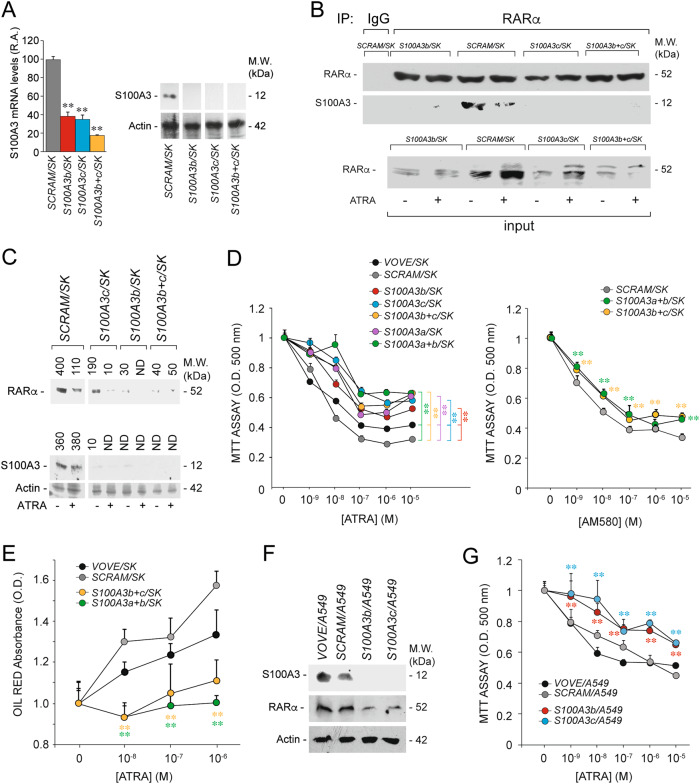
Functional studies in breast cancer *SK-BR-3* and lung cancer *A549* cells stably silenced for the S100A3 gene. **a** *SK-BR-3* cells were stably infected with lentiviruses containing the following short hairpin RNAs: *shSCRAM*, *shS100A3b,* and *shS100A3c* or an equimolar combination of the two lentiviral vectors *shS100A3b* and *shS100A3c*. Infected cells were selected in puromycin for 10 days obtaining the following cell populations: *SCRAM/SK*, *S100A3b/SK*, *S100A3c/SK*, and *S100A3b*+*c/SK*. Left: the bar graph illustrates the relative S100A3 mRNA content determined by RT-PCR on total RNA extracted from the indicated cell populations grown under standard conditions. Each value is the mean ± SD of three independent cell cultures. Right: cell extracts from the indicated cell populations were subjected to western blot analysis using anti-S100A3 and anti-actin antibodies. Each line shows cropped lanes of the same gel, hence the results can be compared across the lanes, as they were obtained with the same exposure time. M.W. = molecular weights of the indicated proteins. **b** The indicated *SK-BR-3* derived cell populations were treated with vehicle (DMSO) or ATRA (1 µM) for 1 h. At the end of the treatment, total cell extracts were immunoprecipitated with anti-RARα mouse monoclonal antibodies (IP: RARα). The negative control for the immunoprecipitations is represented by the extracts challenged with non-specific immuno-globulins G (IP: IgG), as indicated. Following normalization for the content of RARα in the input, the immunoprecipitates were subjected to western blot analysis with anti-RARα rabbit polyclonal antibodies and anti-S100A3 mouse monoclonal antibodies, as indicated. Input = western blot analyses of the cell extracts before the immunoprecipitation step. M.W. = molecular weights of the indicated proteins. **c** The indicated *SK-BR-3* derived cell populations were treated with vehicle (DMSO) or ATRA (1 µM) for 24 h. Cell extracts were subjected to western blot analysis with anti-RARα, anti-S100A3, and anti-actin antibodies. The number above each lane indicates the results obtained after densitometric analysis of the bands and is expressed in arbitrary units. **d** *SK-BR-3* cell populations stably infected with the void lentiviral vector (*VOVE/SK*), the scrambled negative control shRNA (*SCRAM/SK*), the indicated S100A3-targeting shRNA lentiviral constructs and combinations thereof were treated with vehicle and increasing concentrations of ATRA (left) or the RARα agoinist, AM580 (right) for 6 days. The growth of each cell population was evaluated with the use of the MTS assay. Each experimental point is the mean ± S.E. of four independent cell cultures. The MTS absorbance values of the *S100A3b/SK*, *S100A3c/SK,* and *S100A3b*+*c/SK* cell populations exposed to all the indicated concentrations of ATRA are significantly higher than the corresponding values determined for the *VOVE/SK* and *SCRAM/SK* counterparts (*p* < 0.01 according to a two-way Student’s *t*-test). With the exception of *S100A3a*+*b/SK* (10^–6^ M), the MTS absorbance values of the *S100A3a*+*b/SK*, *S100A3b*+*c/SK* cell populations exposed to all the indicated concentrations of AM580 are significantly higher than the corresponding values determined for the *VOVE/SK* and *SCRAM/SK* counterparts (**p < 0.01 according to a two-way Student’s *t*-test). **e** The indicated *SK-BR-3-*derived cell populations were treated with vehicle and increasing concentrations of ATRA for 6 days. The lactogenic differentiation state was determined with the use of the OIL-RED assay. Each experimental point is the mean + S.E. of four independent cell cultures. The OIL-RED absorbance values of the *S100A3a*+*b/SK* and *S100A3b*+*c/SK* cell populations exposed to all the indicated concentrations of ATRA are significantly lower than the corresponding values determined for the *VOVE/SK* and *SCRAM/SK* counterparts (**p < 0.01 according to a two-way Student’s *t*-test). **f** *A549* cells were stably infected with a void lentiviral vector (*VOVE*) or lentiviruses containing *shSCRAM*, *shS100A3b*, and *shS100A3c*. Infected cells were selected in puromycin for 10 days obtaining the following cell populations: *VOVE/A549*, *SCRAM/A549*, *S100A3b/A549*, and *S100A3c/A549*. Cell extracts were subjected to western blot analysis with anti-RARα, anti-S100A3, and anti-actin antibodies. M.W. = molecular weights of the indicated proteins. **g** The indicated *A549* derived cell populations were treated with vehicle and increasing concentrations of ATRA for 3 days. The growth of each cell populations was evaluated with the use of the MTS assay. Each experimental point is the mean ± S.E. of four independent cell cultures. The MTS absorbance values of the *S100A3b/A549* and *S100A3c/A549* cell populations exposed to all the indicated concentrations of ATRA are significantly higher than the corresponding values determined for the *VOVE/A549* and *SCRAM/A549* counterparts (**p < 0.01 according to a two-way Student’s *t*-test)

To evaluate whether modulation of S100A3 expression has any consequence on ATRA anti-tumor activity, we took a silencing approach. We designed three S100A3-targeting shRNAs (*shS100A3-a*, *shS100A3-b,* and *shS100A3-c*) and a scrambled control shRNA (*shSCRAM*). The specificity of our shRNAs is supported by the results obtained in *COS-7* cells over-expressing S100A3 (Supplementary Fig. 7A). We infected *SK-BR-3* cells with *shS100A3-b* and *shS100A3-c* lentiviruses alone or in combination, *shSCRAM* and the void lentivirus (*VOVE*) isolating the following cell populations: *S100A3b/SK*, *S100A3c/SK*, *S100A3b*+*c/SK*, *SCRAM/SK*, and *VOVE/SK*. *S100A3b/SK*, *S100A3c/SK*, and *S100A3b*+*c/SK* express lower levels of S100A3 mRNA/protein than *SCRAM/SK* cells (Fig. 5a). As a consequence, anti-RARα antibodies fail to co-immunoprecipitate S100A3 from S100A3 knockdown cells (Fig. 5b). Consistent with S100A3-dependent inhibition of RARα degradation, *S100A3b/SK*, *S100A3c/SK*, and *S100A3b*+*c/SK* show lower levels of the retinoid-receptor than *SCRAM/SK* cells (Fig. 5c). This effect is evident in basal conditions and following ATRA treatment.

We investigated the consequences of S100A3 silencing on the proliferation of *SK-BR-3* grown under basal conditions. S100A3 knockdown exerts minor and divergent effects on the growth of *SK-BR-3* cells, depending on the *SCRAM/SK* or *VOVE/SK* control line considered (Supplementary Fig. 7B). Given the key-role played by RARα in mediating ATRA anti-proliferative action in breast cancer cells, we also investigated the consequences of S100A3 silencing on ATRA-dependent growth inhibition of *SK-BR-3* cells. Relative to *SCRAM/SK* and *VOVE/SK*, *S100A3a/SK, S100A3a*+*b/SK, S100A3b/SK*, *S100A3c/SK*, and *S100A3b*+*c/SK* cells show decreased sensitivity to the dose-dependent anti-proliferative action of ATRA (Fig. 5d-left). We supported the involvement of RARα, performing experiments in AM580-treated *S100A3a+b/SK*, *S100A3b*+*c/SK*, and *SCRAM/SK* cells. In this case too, S100A3 silencing results in decreased sensitivity to AM580 (Fig. 5d-right).

To establish whether S100A3 silencing affects other aspects of ATRA activity, besides growth inhibition, we studied the lactogenic response [43], by measuring the cellular content of lipids [43] (Fig. 5e). While ATRA causes a dose-dependent increase of lipids in *SCRAM/SK* and *VOVE/SK*, this increase is suppressed in *S100A3a*+*b/SK* and *S100A3b*+*c/SK* cells. Thus, S100A3 knockdown reduces ATRA anti-proliferative and differentiating activity.

To evaluate whether these effects are observed in other cell-lines, we isolated lung cancer *A549* cells infected with *VOVE* (*VOVE/A549*), scrambled shRNA (*SCRAM/A549*), *shS100A3b* (*S100A3b/A549*), and *shS100A3c* (*S100A3c/A549*). *S100A3b/A549* and *S100A3c/A549* cells show the expected knockdown of the S100A3 protein (Fig. 5f). As observed in *SK-BR-3* cells, S100A3 knockdown exerts minor and divergent effects on the basal growth of *A549* cells, depending on the shRNA considered (Supplementary Fig. 7C). Noticeably, silencing of S100A3 in *S100A3b/A549* and *S100A3c/A549* cells decreases the anti-proliferative action of ATRA significantly and consistently (Fig. 5g). This is accompanied by lower levels of RARα than *VOVE/A549* and *SCRAM/A549* controls (Fig. 5f).

### S100A3 controls basal and ATRA-dependent differentiation/proliferation of PML-RARα^+^ NB4 cells

**Fig. 6:**
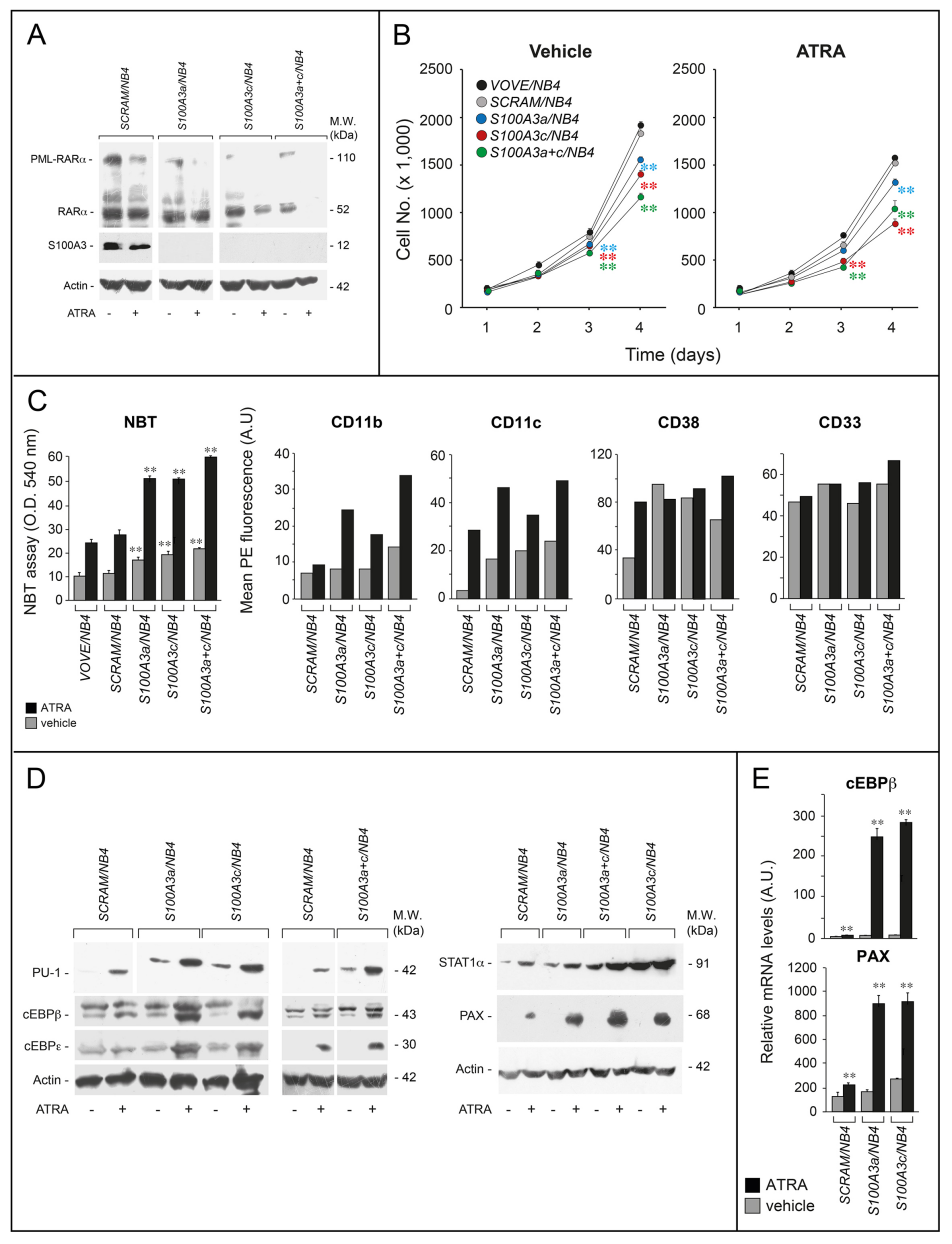
Functional studies in APL-derived *NB4* cells stably silenced for the S100A3 gene. *NB4* cells were stably infected with lentiviruses containing *shSCRAM*, *shS100A3a,* and *shS100A3c* or an equimolar combination of the two lentiviral vectors *shS100A3a* and *shS100A3c*. Infected cells were selected in puromycin for 10 days obtaining the following cell populations: *SCRAM/NB4*, *S100A3a/NB4*, *S100A3c/NB4* and *S100A3a*+*c/NB4*. **a** The indicated *NB4* derived cell populations were treated with vehicle (DMSO) or ATRA (10^–6^ M) for 24 h. Cell extracts were subjected to western blot analysis with anti-RARα, anti-S100A3, and anti-actin antibodies. Each line shows cropped lanes of the same gel, hence the results can be compared across the lanes, as they were obtained with the same exposure time. M.W. = molecular weights of the indicated proteins. **b** The indicated *NB4* derived cell populations were treated with vehicle and ATRA (10^–7^ M) for the indicated amount of time. The growth of each cell population was evaluated by counting the number of viable cells. Each experimental point is the mean ± S.E. of three independent cell cultures. The cell number values of the indicated shRNA expressing cell populations exposed to vehicle or ATRA are significantly lower than the corresponding values determined for the *VOVE/NB4* and *SCRAM/NB4* counterparts (**p < 0.01 according to a two-way Student’s *t*-test). **c** The indicated *NB4* derived cell populations were treated with ATRA (10^–8^ M) for three days. Left: cells were subjected to the NBT assay. Each experimental point is the mean ± S.E. of three independent cell cultures. **The NBT absorbance values of the *S100A3a/NB4*, *S100A3c/NB4*, and *S100A3a*+*c/NB4* cell populations are significantly higher than the corresponding values determined for the *VOVE/NB4* and *SCRAM/NB4* counterparts (*p* < 0.01 according to a two-way Student’s *t*-test). Right: the indicated *NB4* derived cell populations were treated with vehicle or ATRA (10^–8^ M) for 2 days. Cells were subjected to FACS analyses for the CD11b, CD11c, CD38, and CD33 surface markers, as indicated. The results are representative of two other independent experiments. **d** The indicated *NB4* derived cell populations were treated with vehicle or ATRA (10^–7^ M) for 1 day. Cell extracts were subjected to western blot analysis for the indicated proteins using specific antibodies. Each line shows cropped lanes of the same gel, hence the results can be compared across the lanes, as they were obtained with the same exposure time. The results are representative of two other independent experiments. M.W. = molecular weights of the indicated proteins. **e** The indicated cell lines were treated with vehicle (DMSO) or ATRA (1 µM) for 24 h. Total RNA was extracted and subjected to RT-PCR with Taqman assays for the cEBPβ and paxillin. The results are the mean ± SD of three replicates. **Significantly higher relative to the corresponding vehicle-treated sample (Student’s test *p* < 0.01)

S100A3 interacts not only with RARα but also with the APL-specific PML-RARα oncogenic protein. Thus, we evaluated the functional consequences of S100A3 silencing in PML-RARα^+^ and APL-derived *NB4* cells infected with *shS100A3a* (*S100A3a/NB4*), *shS100A3c* (*S100A3c/NB4*), *shS100A3a*+*shS100A3c* (*S100A3a*+*c/NB4*)*, shSCRAM* (*SCRAM/NB4*), and *VOVE* (*VOVE/NB4*) (Fig. 6a). Unlike *SCRAM/NB4*, *S100A3a/NB4*, *S100A3c/NB4,* and *S100A3a*+*c/NB4* cells do not express detectable amounts of S100A3 and show decreased levels of RARα and PML-RARα in basal conditions and more so after ATRA treatment. In addition, *S100A3a/NB4*, *S100A3c/NB4*, and S100A3a+c/NB4 grow more slowly than the *VOVE/NB4* and *SCRAM/NB4* controls (Fig. 6b). Finally, S100A3 knockdown enhances the anti-proliferative effect of ATRA, which is opposite to what is observed in *SK-BR-3* and *A549* cells.

The therapeutic action of ATRA in APL cells is consequent to a reversion of the differentiation block caused by PML-RARα. The phenomenon is recapitulated in *NB4* cells which undergo granulocytic differentiation upon ATRA treatment [30, 31, 37, 44]. The basal levels of the granulocytic differentiation marker, NBT-reducing-activity [45], are higher in *S100A3a/NB4*, *S100A3c/NB4*, and *S100A3a*+*c/NB4* cells than the *SCRAM/NB4* and *VOVE/NB4* controls (Fig. 6c). In addition, ATRA causes a higher increase of NBT-reducing-activity in S100A3-silenced than control cells. S100A3 knockdown results in constitutive upregulation of the other differentiation markers, CD11c (Supplementary Fig. 8A, Fig. 6c) and CD38 (Fig. 6c). At the ATRA concentrations used, the expected induction of CD38 is already maximal in *SCRAM/NB4*, *S100A3a/NB4*, *S100A3c/NB4*, and *S100A3a*+*c/NB4* cells. In contrast, silencing of S100A3 causes a reproducible enhancement of CD11c and CD11b (another differentiation marker) induction by ATRA (Fig. 6c). As expected, CD33 is constitutively expressed in *NB4* cells and its expression is left unaffected by ATRA [4]. Consistent with all this, S100A3 knockdown is associated with morphological features of granulocytic differentiation (increase in the volume of the cytoplasm and the number of granulocytic vesicles), which are already visible under basal conditions and enhanced by ATRA (Supplementary Fig. 8B).

Among the genes induced by ATRA in APL cells [32], the PU-1, cEBPβ, cEBPε, and STAT1 transcription factors as well as the focal adhesion protein, paxillin, are involved in granulocytic differentiation [46, 47]. ATRA induces all these proteins in *SCRAM/NB4* cells and this induction is magnified in *S100A3a/NB4*, *S100A3c/NB4*, and *S100A3a*+*c/NB4* cells (Fig. 6d). Interestingly, S100A3 knockdown elevates the constitutive amounts of PU-1 and STAT1, which may explain the granulocytic-differentiation signs observed under basal conditions. As cEBPβ and paxillin are encoded by direct RAR target genes, we evaluated whether the increase in ATRA-dependent upregulation by S100A3 knockdown is consistent with a transcriptional effect. Hence, we determined cEBPβ and paxillin mRNA levels in vehicle and ATRA-treated *SCRAM/NB4*, *S100A3a/NB4*, *S100A3c/NB4* cells (Fig. 6e). The results obtained recapitulate what is observed at the protein level, demonstrating that S100A3 knockdown causes a substantial increase in ATRA-dependent induction of the two transcripts. The data support a transcriptional effect mediated by PML-RARα or RARα activation by the retinoid.

**Fig. 7:**
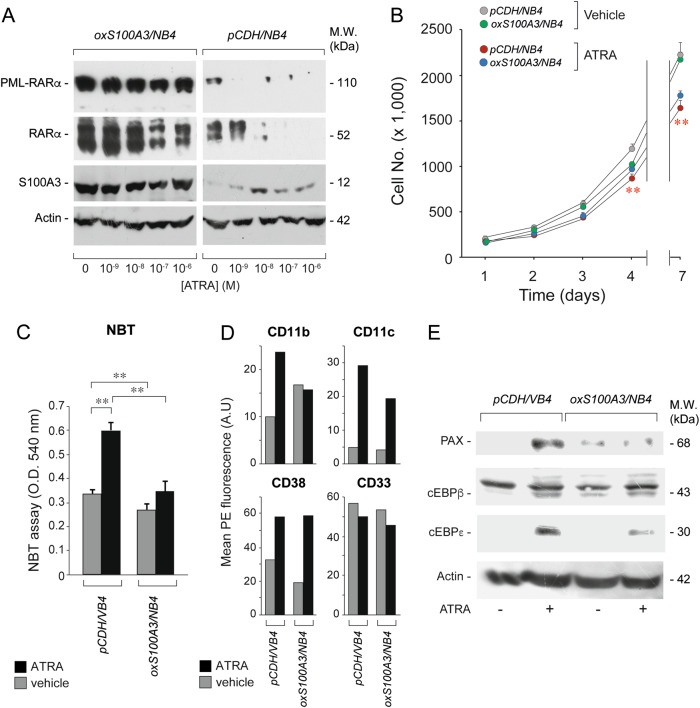
Functional studies in APL-derived *NB4* cells stably over-expressing the S100A3 gene. *NB4* cells were stably infected with a void lentivirus vector (*pCDH-CMV*) or the same lentivirus containing the S100A3 full-length cDNA (*S100A3*). Transfected cells were selected in puromycin for 10 days obtaining the following cell populations: *pCDH/NB4* and *S100ox/NB4*. **a** Cells were treated vehicle (DMSO) and the indicated concentrations of ATRA for 24 h. *pCDH/NB4* and *S100ox/NB4* cell extracts were subjected to western blot analysis with anti-RARα, anti-S100A3 and anti-actin antibodies. M.W. = molecular weights of the indicated proteins. **b** The indicated *NB4* derived cell populations were treated with vehicle and ATRA (10^–7^ M) for up to 7 days. The growth of each cell populations was evaluated by counting the number of viable cells. Each experimental point is the mean ± S.E. of three independent cell cultures. **Significantly lower relative to the corresponding ATRA-treated *pCDH/NB4* controls (Student’s test *p* < 0.01). **c** *pCDH/NB4* and *S100ox/NB4* cells were treated with ATRA (10^–8^ M) for 3 days. Cells were subjected to the NBT assay. Each experimental point is the mean ± S.E. of three independent cell cultures. **Significantly different (*p* < 0.01 according to a two-way Student’s *t*-test). **d** *pCDH/NB4* and *S100ox/NB4* cells were treated with ATRA (10^–8^ M) for 2 days. Cells were subjected to FACS analyses for the CD11b, CD11c, CD38, and CD33 surface markers, as indicated. The results are representative of two other independent experiments. **e** *pCDH/NB4* and *S100ox/NB4* cells were treated with vehicle or ATRA (10^–7^ M) for 1 day. Cell extracts were subjected to western blot analysis for the indicated proteins using specific antibodies. M.W. = molecular weights of the indicated proteins. The results are representative of two other independent experiments

To confirm the results, we used a specular approach and we stably transfected S100A3 and a void vector in *NB4* blasts obtaining *oxS100A3/NB4* and *pCDH/NB4* cells, respectively. S100A3 overexpression is associated with a consistent increase in the levels of both PML-RARα and RARα (Fig. 7a). This is accompanied by inhibition of the ATRA-dependent degradation of the two retinoid-receptors and a modest, though significant, reduction in the anti-proliferative effect of ATRA at 4 and 7 days (Fig. 7b). As for the differentiation state, the basal expression levels of NBT-reducing activity, CD11b, CD11c, CD38, and CD33 in *oxS100A3/NB4* and *pCDH/NB4* cells are similar (Fig. 7c, d). In contrast, *oxS100A3/NB4* cells are characterized by reduced ATRA-dependent upregulation of NBT-reducing activity (Fig. 7c), CD11b, CD11c, cEBPβ, cEBPε, and PAX relative to the corresponding *pCDH/NB4* control (Fig. 7d, e).

### S100A3 knockdown controls ATRA-dependent differentiation/proliferation of PML-RARα^−^ HL-60 cells

**Fig. 8:**
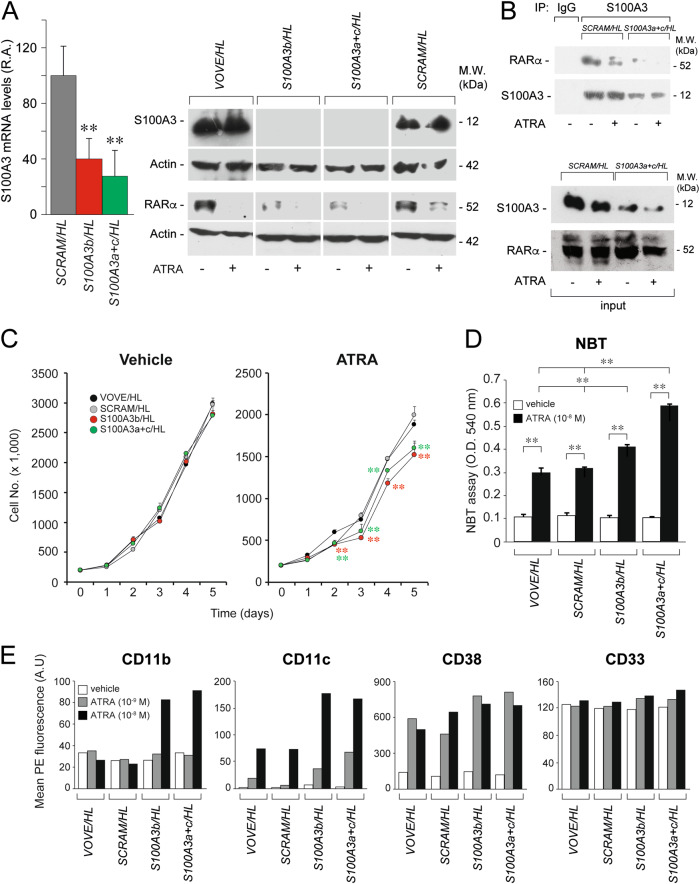
Functional studies in *HL-60* cells stably silenced for the S100A3 gene. *HL-60* cells were stably infected with lentiviruses containing *shSCRAM*, *shS100A3b* and an equimolar combination of the two lentiviral vectors *shS100A3a*+*shS100A3c* as well as the corresponding void lentivirus (VOVE). Infected cells were selected in puromycin for 10 days obtaining the following cell populations: *VOVE/HL*, *SCRAM/HL*, *S100A3b/HL,* and *S100A3a*+*c/HL*. **a** The indicated *HL-60* derived cell populations were treated with vehicle (DMSO) or ATRA (10^–6^ M) for 24 h. Cell extracts were subjected to western blot analysis with anti-S100A3, anti-RARα and anti-actin antibodies. Each line shows cropped lanes of the same gel, hence the results can be compared across the lanes, as they were obtained with the same exposure time. M.W. = molecular weights of the indicated proteins. **b** *SCRAM/HL* and *S100A3a*+*c/HL* cells were treated with vehicle (DMSO) or ATRA (1 µM) for 1 h. At the end of the treatment, total cell extracts were immunoprecipitated with anti-S100A3 mouse monoclonal antibodies (IP: S100A3). The negative control for the immunoprecipitations is represented by the extracts challenged with non-specific immuno-globulins G (IP: IgG), as indicated. Following normalization for the content of S100A3 in the input, the immunoprecipitates were subjected to western blot analysis with anti-RARα and anti-S100A3 antibodies, as indicated. Input = western blot analyses of the cell extracts before the immunoprecipitation step. M.W. = molecular weights of the indicated proteins. **c** The indicated *HL-60* derived cell populations were treated with vehicle and ATRA (10^–7^ M) for the indicated amount. The growth of each cell populations was evaluated by counting the number of viable cells. Each experimental point is the mean ± S.E. of three independent cell cultures. The cell number values of the indicated shRNA expressing cell populations exposed to vehicle or ATRA are significantly lower than the corresponding values determined for the *VOVE/HL* and *SCRAM/HL* counterparts (**p < 0.01 according to a two-way Student’s *t*-test). **d** The indicated *HL-60* derived cell populations were treated with ATRA (10^–8^ M) for 5 days. Cells were subjected to the NBT assay. Each experimental point is the mean ± S.E. of three independent cell cultures. **Significantly higher (*p* < 0.01 according to a two-way Student’s *t*-test). **e** The indicated *HL-60* derived cell populations were treated with vehicle or ATRA (10^–8^ M and 10^–9^ M) for 2 days. Cells were subjected to FACS analyses for the CD11b, CD11c, CD38, and CD33 surface markers, as indicated. The results are representative of two other independent experiments

To establish the relevance of the cellular context and/or PML-RARα expression for the different effects afforded by S100A3 silencing in *NB4* and *SK-BR-3/A549*, we considered the *HL-60* AML cell line. *HL-60* cells do not express PML-RARα, undergo granulocytic differentiation upon exposure to ATRA [48] and contain measurable levels of S100A3, which interacts with RARα (see *VOVE/HL* and *SCRAM/HL* cells, Fig. 8a, b). We generated two cell populations of *HL-60* cells stably silenced for S100A3 (*S100A3b/HL*; *S100A3a*+*c/HL*) and two control populations (*SCRAM/HL; VOVE/HL*) (Fig. 8a). As observed in *NB4* cells, S100A3 silencing was associated with decreased steady-state levels of the RARα protein. Unlike the *NB4* counterpart, S100A3 knockdown does not affect the basal growth (Fig. 8c) differentiation state (NBT assay, CD11b, CD11c, and CD38 expression) of *HL-60* cells (Fig. 8d, e). In contrast, S100A3 silencing enhances ATRA growth-inhibitory and differentiating effects. As the results obtained in S100A3-silenced *HL-60* and *NB4* cells exposed to ATRA are substantially similar, our data indicate that the cellular context rather than PML-RARα expression is the major determinant of the different effects exerted by S100A3 on RARα functional activity in myeloid leukemia relative to breast cancer and lung cancer cells.

## Discussion

The study reports on the identification of novel proteins interacting with RARα in breast cancer cells [3]. Among the identified proteins, we focused our attention on S100A3, a member of the S100 family of calcium-binding proteins [49, 50]. There are no data regarding the potential involvement of S100A3 in the retinoid signal transduction pathway, although other members of the family, i.e. S100A9 and S100A10, have been implicated in ATRA-induced differentiation of APL cells [51–54]. Our data indicate that S100A3 binds to unliganded RARα directly and binding is reduced by ATRA. The H12-helix located in the ligand-binding E-region of RARα plays a crucial role in the interaction with S100A3. In particular, the I396 residue, laying in proximity to the H12-helix is of fundamental importance. The H12-helix and I396 are conserved in RARβ and RARγ, yet only RARγ interacts with S100A3. In S100A3, a sequence similar to the one involved in the binding of co-repressor molecules to RARs (Supplementary Fig. 4) is likely to underlay the ability to compete with the N-COR co-repressor for the binding to RARα. Our data are consistent with the idea that S100A3 may act as a RARα co-repressor and suggest that S100A3 and N-COR are unlikely to be part of the same co-repressor complex. The RARα/S100A3 interaction is cell-context independent and it occurs predominantly in the cell-nucleus.

From a functional point of view, our results support the concept that the dynamic interaction with S100A3 is involved in the control of constitutive and ATRA-dependent RARα degradation in a cell-context independent manner. Similar effects are observed in the case of the APL-specific PML-RARα fusion protein. The studies performed in S100A3 over-expressing *COS-7*, *SK-BR-3* (MG, unpublished results), and *NB4* cells demonstrate an increase in the constitutive steady-state levels of both RARα and PML-RARα, due to a decrease in the proteasome-dependent degradation of the two proteins. The inhibitory effect on RARα and PML-RARα degradation by S100A3 is associated with a reduction in the ligand-dependent transcriptional activity of the two retinoid-receptors. This is the opposite of what is observed upon interaction of the p38 MAP-kinase with both receptors [37]. In fact, binding of p38 MAP-kinase to RARα and PML-RARα causes an increase in the degradation and a decrease in the transcriptional activity of the two receptors. The above observations indicate that proteasome-dependent degradation is a crucial determinant of RARα and PML-RARα function, although its inhibition is not necessarily associated with an increase in their transcriptional activity [37].

The control exerted by S100A3 on RARα modulates the anti-tumor activity of ATRA in breast cancer *SK-BR-3* and lung cancer *A549* cells. Here, S100A3 knockdown reduces the response to the anti-proliferative action of ATRA. In *SK-BR-3* cells a similar effect is observed also as far as the retinoid differentiating activity. Induction of ATRA-resistance is consistent with the primary role exerted by RARα in mediating the anti-tumor action of the retinoid in breast cancer [3] and the decrease in the levels of RARα afforded by S100A3 knockdown. Interestingly, opposite effects are observed in PML-RARα^+^ *NB4* and PML-RARα^−^ *HL-60* AML cells, where S100A3 knockdown increases the ATRA-dependent anti-proliferative and differentiating responses, despite down-regulation of RARα. In *NB4* cells, S100A3 silencing induces not only ATRA-dependent but also basal differentiation. This is consistent with PML-RARα down-regulation by S100A3 silencing. Indeed, unliganded PML-RARα is believed to be responsible for the differentiation arrest of the APL blast and decreased expression of the translocation product by S100A3 knockdown may at least partially release the differentiation block. Noticeably, this is the mechanism proposed for the potentiating action of arsenic trioxide on ATRA in APL [1, 2]. In *NB4* cells, S100A3 knockdown seems to cause a larger reduction in the levels of PML-RARα than RARα, altering the relative abundance of the two proteins in favor of the latter one. This may release residual RARα from the dominant-negative effect exerted by the oncogenic protein, potentiating the ATRA growth-inhibitory/differentiating effects. However, other myeloid-cell specific mechanisms must also be involved in the enhancement of the ATRA-dependent anti-proliferative effect afforded by S100A3 silencing, as indicated by the results obtained in PML-RARα^−^ and AML-derived *HL-60* cells.

In conclusion, the study demonstrates that S100A3 interacts with and is involved in the proteasomal-dependent degradation of RARα. Our results provide new insights on the molecular mechanisms underlying the control of RARα functional activity and may have practical implications. In fact, S100A3 represents a novel pharmacological target for the development of rational drug combinations aimed at potentiating the therapeutic activity of ATRA.

## Materials and methods

### Interactomic studies

The proteomics data were deposited in the ProteomeXchange Consortium *via* the PRIDE partner repository with the dataset identifier PXD00876 and further methodological details are available in Supplementary Methods.

### Cells and infection procedures

The source of the cell lines and the infection procedures are described in Supplementary Methods. We generated *SK-BR-3, A549, HL-60*, and *NB4* cell populations silenced for S100A3 with lentiviral vectors (pGREENpuro, System Biosciences) containing the shRNA. To isolate *NB4* populations over-expressing S100A3, cells were infected with *pCDH-CMV* lentiviral vectors (System Biosciences) containing the human S100A3 cDNA. To this purpose, S100A3 was inserted in the XbaI and NotI sites of *pCDH-CMV*.

### Transient overexpression and transactivation studies in COS-7 cells

*COS-7* cells were transiently co-transfected with an expression plasmid for the S100A3 cDNA (S100A3) or the corresponding void vector (*pcDNA3*) in the presence of *pSG5* plasmids containing RARα, PML-RARα, RARα2, PML or the RARα point as well as deletion-mutants described in Supplementary Figure 4. The methodologies used are as described [32].

### Immunoprecipitation, far-western, GST pull-down assays, FACS analysis, immunoprecipitation, and western blot analyses

Immunoprecipitation, far-western, and GST pull-down assays were performed in *COS-7* cells using already described approaches and methodologies [32]. Further details are available in Supplementary Methods. CD11b, CD11c, CD38, and CD33 markers were determined with a fluorescence activated cell sorter (FACS, Becton and Dickinson) [29, 37]. Western blot analyses were performed as previously described [29, 32, 37]. Agarose beads coupled to anti-HA antibodies were from Sigma (A2095).

## Supplementary information

Supplemental Material

## References

[1] de The H (2018). Differentiation therapy revisited. Nat Rev Cancer.

[2] de The H, Pandolfi PP, Chen Z (2017). Acute promyelocytic leukemia: a paradigm for oncoprotein-targeted cure. Cancer Cell.

[3] Centritto F, Paroni G, Bolis M, Garattini SK, Kurosaki M, Barzago MM (2015). Cellular and molecular determinants of all-trans retinoic acid sensitivity in breast cancer: luminal phenotype and RARα expression. EMBO Mol Med.

[4] Gianni M, Li Calzi M, Terao M, Guiso G, Caccia S, Barbui T (1996). AM580, a stable benzoic derivative of retinoic acid, has powerful and selective cyto-differentiating effects on acute promyelocytic leukemia cells. Blood.

[5] Bolis M, Garattini E, Paroni G, Zanetti A, Kurosaki M, Castrignano T, et al. Network-guided modelling allows tumor-type independent prediction of sensitivity to all-trans retinoic acid. Ann Oncol. 2016;28:611–21. 10.1093/annonc/mdw660 PMC5834014 27993792

[6] Chambon P (1996). A decade of molecular biology of retinoic acid receptors. FASEB J.

[7] Germain P, Chambon P, Eichele G, Evans RM, Lazar MA, Leid M (2006). International union of pharmacology. LXIII. Retinoid X receptors. Pharmacol Rev.

[8] Germain P, Chambon P, Eichele G, Evans RM, Lazar MA, Leid M (2006). International union of pharmacology. LX. Retinoic acid receptors. Pharmacol Rev.

[9] Benbrook DM, Chambon P, Rochette-Egly C, Asson-Batres MA (2014). History of retinoic acid receptors. Subcell Biochem.

[10] Mark M, Ghyselinck NB, Chambon P (2006). Function of retinoid nuclear receptors: lessons from genetic and pharmacological dissections of the retinoic acid signaling pathway during mouse embryogenesis. Annu Rev Pharmacol Toxicol.

[11] Lalevee S, Ferry C, Rochette-Egly C (2010). Phosphorylation control of nuclear receptors. Methods Mol Biol.

[12] Zhu J, Gianni M, Kopf E, Honore N, Chelbi-Alix M, Koken M (1999). Retinoic acid induces proteasome-dependent degradation of retinoic acid receptor alpha (RARα) and oncogenic RARα fusion proteins. Proc Natl Acad Sci USA.

[13] Sahin U, Lallemand-Breitenbach V, de The H (2014). PML nuclear bodies: regulation, function and therapeutic perspectives. J Pathol.

[14] Bolis M, Garattini E, Paroni G, Zanetti A, Kurosaki M, Castrignano T (2017). Network-guided modeling allows tumor-type independent prediction of sensitivity to all-trans-retinoic acid. Ann Oncol.

[15] Hoedt E, Chaoui K, Huvent I, Mariller C, Monsarrat B, Burlet-Schiltz O (2014). SILAC-based proteomic profiling of the human MDA-MB-231 metastatic breast cancer cell line in response to the two antitumoral lactoferrin isoforms: the secreted lactoferrin and the intracellular delta-lactoferrin. PLoS ONE.

[16] Kani K (2017). Quantitative proteomics using SILAC. Methods Mol Biol.

[17] Joo K, Kim CG, Lee MS, Moon HY, Lee SH, Kim MJ (2013). CCDC41 is required for ciliary vesicle docking to the mother centriole. Proc Natl Acad Sci USA.

[18] Li XP, Jiao JU, Lu LI, Zou Q, Zhu S, Zhang Y (2016). Overexpression of ribosomal L1 domain containing 1 is associated with an aggressive phenotype and a poor prognosis in patients with prostate cancer. Oncol Lett.

[19] Eckert RL, Broome AM, Ruse M, Robinson N, Ryan D, Lee K (2004). S100 proteins in the epidermis. J Invest Dermatol.

[20] Heizmann CW, Ackermann GE, Galichet A (2007). Pathologies involving the S100 proteins and RAGE. Subcell Biochem.

[21] Hu L, Bikle DD, Oda Y (2014). Reciprocal role of vitamin D receptor on beta-catenin regulated keratinocyte proliferation and differentiation. J Steroid Biochem Mol Biol.

[22] Berry DC, Noy N (2007). Is PPARβ/δ a retinoid receptor?. PPAR Res.

[23] Levi L, Wang Z, Doud MK, Hazen SL, Noy N (2015). Saturated fatty acids regulate retinoic acid signalling and suppress tumorigenesis by targeting fatty acid-binding protein 5. Nat Commun.

[24] Schug TT, Berry DC, Shaw NS, Travis SN, Noy N (2007). Opposing effects of retinoic acid on cell growth result from alternate activation of two different nuclear receptors. Cell.

[25] Caccamo D, Condello S, Ferlazzo N, Curro M, Griffin M, Ientile R (2013). Transglutaminase 2 interaction with small heat shock proteins mediate cell survival upon excitotoxic stress. Amino Acids.

[26] Hatakeyama D, Kozawa O, Niwa M, Matsuno H, Ito H, Kato K (2002). Upregulation by retinoic acid of transforming growth factor-beta-stimulated heat shock protein 27 induction in osteoblasts: involvement of mitogen-activated protein kinases. Biochim Biophys Acta.

[27] Steinbach D, Pfaffendorf N, Wittig S, Gruhn B (2007). PRAME expression is not associated with down-regulation of retinoic acid signaling in primary acute myeloid leukemia. Cancer Genet Cytogenet.

[28] Danielpour D, Kim KY, Winokur TS, Sporn MB (1991). Differential regulation of the expression of transforming growth factor-beta s 1 and 2 by retinoic acid, epidermal growth factor, and dexamethasone in NRK-49F and A549 cells. J Cell Physiol.

[29] Gianni M, Boldetti A, Guarnaccia V, Rambaldi A, Parrella E, Raska I (2009). Inhibition of the peptidyl-prolyl-isomerase Pin1 enhances the responses of acute myeloid leukemia cells to retinoic acid via stabilization of RARα and PML-RARα. Cancer Res.

[30] Gianni M, Kalac Y, Ponzanelli I, Rambaldi A, Terao M, Garattini E (2001). Tyrosine kinase inhibitor STI571 potentiates the pharmacologic activity of retinoic acid in acute promyelocytic leukemia cells: effects on the degradation of RARα and PML-RARα. Blood.

[31] Gianni M, Fratelli M, Bolis M, Kurosaki M, Zanetti A, Paroni G (2017). RARα2 and PML-RAR similarities in the control of basal and retinoic acid induced myeloid maturation of acute myeloid leukemia cells. Oncotarget.

[32] Gianni M, Fratelli M, Bolis M, Kurosaki M, Zanetti A, Paroni G (2016). RARα2 and PML-RAR similarities in the control of basal and retinoic acid induced myeloid maturation of acute myeloid leukemia cells. Oncotarget.

[33] Piskunov A, Al Tanoury Z, Rochette-Egly C (2014). Nuclear and extra-nuclear effects of retinoid acid receptors: how they are interconnected. Subcell Biochem.

[34] Gianni M, Parrella E, Raska I, Gaillard E, Nigro EA, Gaudon C (2006). P38MAPK-dependent phosphorylation and degradation of SRC-3/AIB1 and RARα-mediated transcription. EMBO J.

[35] Gaillard E, Bruck N, Brelivet Y, Bour G, Lalevee S, Bauer A (2006). Phosphorylation by PKA potentiates retinoic acid receptor alpha activity by means of increasing interaction with and phosphorylation by cyclin H/cdk7. Proc Natl Acad Sci USA.

[36] Tahayato A, Lefebvre P, Formstecher P, Dautrevaux M (1993). A protein kinase C-dependent activity modulates retinoic acid-induced transcription. Mol Endocrinol.

[37] Gianni M, Peviani M, Bruck N, Rambaldi A, Borleri G, Terao M (2012). p38αMAPK interacts with and inhibits RARα: suppression of the kinase enhances the therapeutic activity of retinoids in acute myeloid leukemia cells. Leukemia.

[38] Yoshida H, Kitamura K, Tanaka K, Omura S, Miyazaki T, Hachiya T (1996). Accelerated degradation of PML-retinoic acid receptor alpha (PML-RARA) oncoprotein by all-trans-retinoic acid in acute promyelocytic leukemia: possible role of the proteasome pathway. Cancer Res.

[39] Carrier M, Lutzing R, Gaouar S, Rochette-Egly C (2018). TRIM24 mediates the interaction of the retinoic acid receptor alpha with the proteasome. FEBS Lett.

[40] Hu X, Lazar MA (1999). The CoRNR motif controls the recruitment of corepressors by nuclear hormone receptors. Nature.

[41] le Maire A, Teyssier C, Erb C, Grimaldi M, Alvarez S, de Lera AR (2010). A unique secondary-structure switch controls constitutive gene repression by retinoic acid receptor. Nat Struct Mol Biol.

[42] Soderstrom M, Vo A, Heinzel T, Lavinsky RM, Yang WM, Seto E (1997). Differential effects of nuclear receptor corepressor (N-CoR) expression levels on retinoic acid receptor-mediated repression support the existence of dynamically regulated corepressor complexes. Mol Endocrinol.

[43] Paroni G, Fratelli M, Gardini G, Bassano C, Flora M, Zanetti A (2012). Synergistic antitumor activity of lapatinib and retinoids on a novel subtype of breast cancer with coamplification of ERBB2 and RARA. Oncogene.

[44] Garattini E, Gianni M, Terao M (2007). Cytodifferentiation by retinoids, a novel therapeutic option in oncology: rational combinations with other therapeutic agents. Vitam Horm.

[45] Parrella E, Gianni M, Cecconi V, Nigro E, Barzago MM, Rambaldi A (2004). Phosphodiesterase IV inhibition by piclamilast potentiates the cytodifferentiating action of retinoids in myeloid leukemia cells. Cross-talk between the cAMP and the retinoic acid signaling pathways. J Biol Chem.

[46] Ovcharenko A, Granot G, Shpilberg O, Raanani P (2013). Retinoic acid induces adhesion and migration in NB4 cells through Pyk2 signaling. Leuk Res.

[47] Luscinskas FW (2012). FAK and PAX-illin get involved in leukocyte diapedesis. Eur J Immunol.

[48] Breitman TR, Selonick SE, Collins SJ (1980). Induction of differentiation of the human promyelocytic leukemia cell line (HL-60) by retinoic acid. Proc Natl Acad Sci USA.

[49] Kizawa K, Jinbo Y, Inoue T, Takahara H, Unno M, Heizmann CW (2013). Human S100A3 tetramerization propagates Ca(2+)/Zn(2+) binding states. Biochim Biophys Acta.

[50] Kizawa K, Takahara H, Unno M, Heizmann CW (2011). S100 and S100 fused-type protein families in epidermal maturation with special focus on S100A3 in mammalian hair cuticles. Biochimie.

[51] Wu Y, Mou Z, Li J, Zhou W, Wei B, Zou L (2004). Identification of a S100 calcium-binding protein expressed in HL-60 cells treated with all-trans retinoic acid by two-dimensional electrophoresis and mass spectrometry. Leuk Res.

[52] Zhu Y, Zhang F, Zhang S, Deng W, Fan H, Wang H (2017). Regulatory mechanism and functional analysis of S100A9 in acute promyelocytic leukemia cells. Front Med.

[53] Huang D, Yang Y, Sun J, Dong X, Wang J, Liu H (2017). Annexin A2-S100A10 heterotetramer is upregulated by PML/RARα fusion protein and promotes plasminogen-dependent fibrinolysis and matrix invasion in acute promyelocytic leukemia. Front Med.

[54] O’Connell PA, Madureira PA, Berman JN, Liwski RS, Waisman DM (2011). Regulation of S100A10 by the PML-RAR-α oncoprotein. Blood.

