# Correction: S100A3 a partner protein regulating the stability/activity of RARα and PML-RARα in cellular models of breast/lung cancer and acute myeloid leukemia

**DOI:** 10.1038/s41388-022-02564-8

**Published:** 2022-12-07

**Authors:** Maurizio Gianni, Mineko Terao, Mami Kurosaki, Gabriela Paroni, Laura Brunelli, Roberta Pastorelli, Adriana Zanetti, Monica Lupi, Andrea Acquavita, Marco Bolis, Maddalena Fratelli, Cecile Rochette-Egly, Enrico Garattini

**Affiliations:** 1 Laboratory of Molecular Biology, Istituto di Ricerche Farmacologiche Mario Negri IRCCS, via La Masa 19, 20156 Milano, Italy; 2 Laboratory of Mass Spectrometry, Istituto di Ricerche Farmacologiche Mario Negri IRCCS, via La Masa 19, 20156 Milano, Italy; 3 Laboratory of Cancer Pharmacology, Istituto di Ricerche Farmacologiche Mario Negri IRCCS, via La Masa 19, 20156 Milano, Italy; 4 Department of Functional Genomics and Cancer, IGBMC (Institut de Génétique et de Biologie Moléculaire et Cellulaire), INSERM, U964; CNRS, UMR7104, Université de Strasbourg, 1 rue Laurent Fries, BP 10142, 67404 Illkirch Cedex, France

Correction to: *Oncogene* 10.1038/s41388-018-0599-z, published online 07 December 2018

Following the publication of this article, the authors noted mounting errors in Figures 2C and 6A. Corrected versions of both figures are provided below. It should be noted that the lanes involved in Figure 6a are devoid of any S100A3 signal. The authors confirm these errors have no influence on the interpretation of published results.

**Fig. 2:**
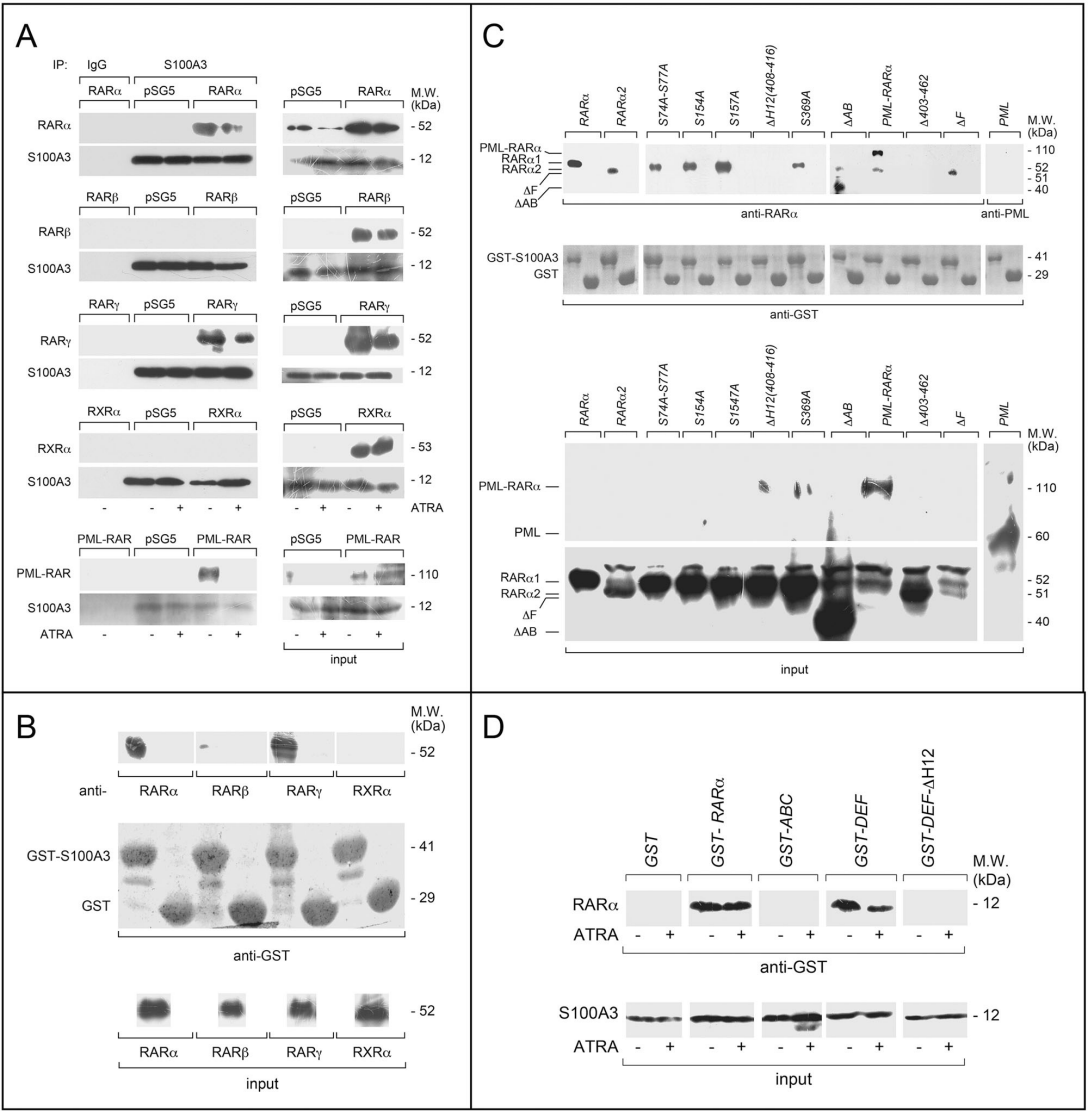
Specificity and structural determinants of RARα binding to S100A3. a COS-7 cells were co-transfected with equal amounts of RARα, PML-RARα, RARβ, RARγ or RXRα, and S100A3 expression plasmids, as indicated. The negative control for the experiments is represented by cells co-transfected with the void expression plasmid (pSG5). Twenty-four hours following transfection, cells were treated with vehicle (DMSO) or ATRA (1 μM) for 1 h. At the end of the treatment, total cell extracts were immunoprecipitated with anti-S100A3 mouse monoclonal antibodies (IP: S100A3). A further negative control for the immunoprecipitations is represented by the extracts of COS-7 cells co-transfected with pSG5 and the S100A3 expression plasmid which were challenged with non-specific immunoglobulins G (IP: IgG). Following normalization for the content of S100A3 in the input, the various immunoprecipitates were subjected to western blot analysis with anti-RARα, anti-RARβ, anti-RARγ, or anti-RXRα antibodies. All the blots were subsequently re-challenged with anti-S100A3 antibodies, as indicated. Input = western blot analysis of the cell extracts before the indicated immunoprecipitation step. Each immunoprecipitation is representative of at least two independent experiments providing the same type of results. **b**, **c** GST pull-down: the GST-tagged recombinant protein, GST-S100A3, and the GST negative control were used. The two recombinant proteins conjugated to Glutathione-Sepharose beads were incubated with extracts of COS-7 cells transfected with the pSG5 expression plasmids containing wildtype RARα, RARβ, RARγ, RXRα, RARα2, PML-RARα and the indicated RARα deletion-mutants and point-mutants. GST pull-down precipitates were blotted on nitro-cellulose filters, incubated with an anti-RARα, anti-RARβ, anti-RARγ, anti-RXRα antibodies (**b**) or anti-RARα antibodies only (**c**). Subsequently the filters were re-blotted with an anti-GST antibody, as indicated. Input: cell extracts (15 μg of protein) representing 10% of the total amount of protein were subjected to western blot analysis with the above anti-RARα antibody. **d** Far-western: COS-7 cells were transfected with the same S100A3 expression plasmid as in (**a**). Cell extracts were precipitated with sepharose beads conjugated with an anti-S100A3 monoclonal antibody. The immunoprecipitates were subjected to far-western analysis using the following GST-tagged RARα recombinant proteins: GSTRARα = full-length RARα; GST-ABC = RARα ABC regions; GSTDEF = RARα DEF regions; GST-DEFΔH12 = RARα1 DEF regions lacking the H12-helix. The blots were developed with an anti-GST antibody. Input: cell extracts (15 μg of protein) representing 10% of the total amount of protein used for the immunoprecipitations were subjected to western blot analysis with an anti-S100A3 antibody. Each line shows cropped lanes of the same gel, hence the results can be compared across the lanes, as they were obtained with the same exposure time. M.W. molecular weights of the indicated proteins.

**Fig. 6:**
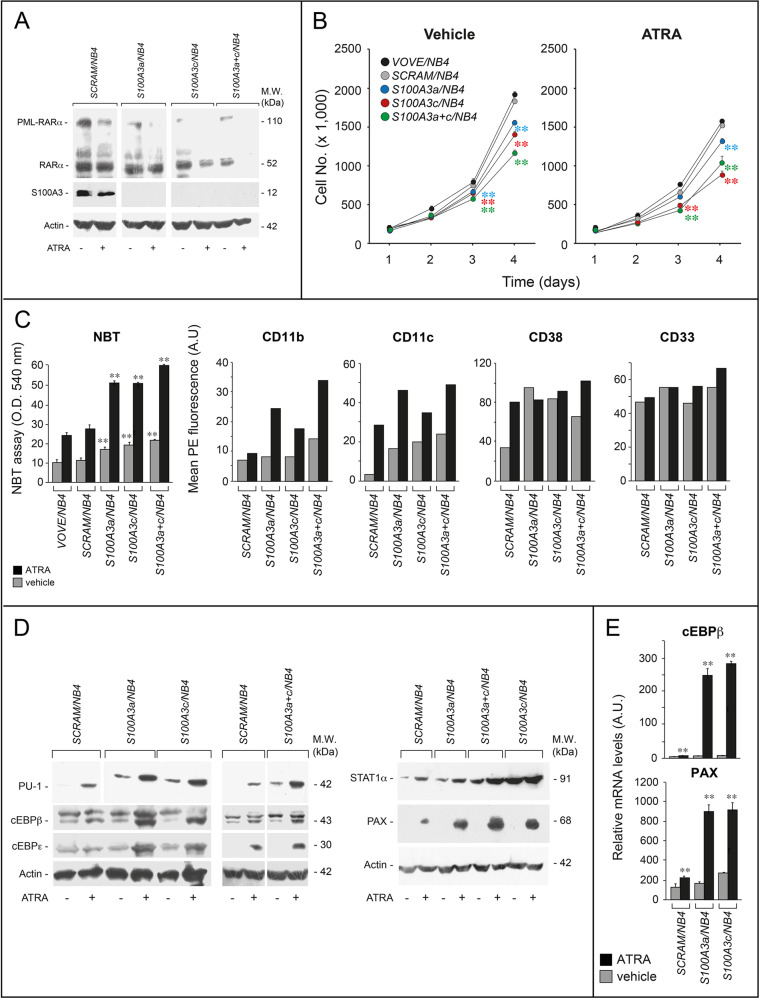
Functional studies in APL-derived NB4 cells stably silenced for the S100A3 gene. NB4 cells were stably infected with lentiviruses containing shSCRAM, shS100A3a, and shS100A3c or an equimolar combination of the two lentiviral vectors shS100A3a and shS100A3c. Infected cells were selected in puromycin for 10 days obtaining the following cell populations: SCRAM/NB4, S100A3a/NB4, S100A3c/ NB4 and S100A3a+c/NB4. **a** The indicated NB4 derived cell populations were treated with vehicle (DMSO) or ATRA (10–6 M) for 24 h. Cell extracts were subjected to western blot analysis with anti-RARα, anti-S100A3, and anti-actin antibodies. Each line shows cropped lanes of the same gel, hence the results can be compared across the lanes, as they were obtained with the same exposure time. M.W. = molecular weights of the indicated proteins. **b** The indicated NB4 derived cell populations were treated with vehicle and ATRA (10–7 M) for the indicated amount of time. The growth of each cell population was evaluated by counting the number of viable cells. Each experimental point is the mean ± S.E. of three independent cell cultures. The cell number values of the indicated shRNA expressing cell populations exposed to vehicle or ATRA are significantly lower than the corresponding values determined for the VOVE/NB4 and SCRAM/NB4 counterparts (**p < 0.01 according to a two-way Student’s *t*-test). **c** The indicated NB4 derived cell populations were treated with ATRA (10–8 M) for three days. Left: cells were subjected to the NBT assay. Each experimental point is the mean ± S.E. of three independent cell cultures. **The NBT absorbance values of the S100A3a/NB4, S100A3c/NB4, and S100A3a+c/NB4 cell populations are significantly higher than the corresponding values determined for the VOVE/NB4 and SCRAM/NB4 counterparts (*p* < 0.01 according to a two-way Student’s *t*-test). Right: the indicated NB4 derived cell populations were treated with vehicle or ATRA (10–8 M) for 2 days. Cells were subjected to FACS analyses for the CD11b, CD11c, CD38, and CD33 surface markers, as indicated. The results are representative of two other independent experiments. **d** The indicated NB4 derived cell populations were treated with vehicle or ATRA (10–7 M) for 1 day. Cell extracts were subjected to western blot analysis for the indicated proteins using specific antibodies. Each line shows cropped lanes of the same gel, hence the results can be compared across the lanes, as they were obtained with the same exposure time. The results are representative of two other independent experiments. M.W. = molecular weights of the indicated proteins. e The indicated cell lines were treated with vehicle (DMSO) or ATRA (1 μM) for 24 h. Total RNA was extracted and subjected to RT-PCR with Taqman assays for the cEBPβ and paxillin. The results are the mean ± SD of three replicates. **Significantly higher relative to the corresponding vehicle-treated sample (Student’s test *p* < 0.01).

