# Supplementary material for: S100A3 a partner protein regulating the stability/activity of RARα and PML-RARα in cellular models of breast/lung cancer and acute myeloid leukemia
Source: Oncogene. 2018 Dec 7;38(14):2482–500. doi: 10.1038/s41388-018-0599-z (PMC6484772; doi:10.1038/s41388-018-0599-z)
Supplement: Supplementary file 1 — Supplemental Material [file 41388_2018_599_MOESM1_ESM.docx]

**Title**: S100A3 a partner protein regulating the stability/activity of RARα and PML-RARα in cellular models of breast/lung cancer and acute myeloid leukemia

Maurizio Gianni’, Mineko Terao, Mami Kurosaki, Gabriela Paroni, Laura Brunelli, Roberta Pastorelli, Adriana Zanetti, Monica Lupi, Andrea Acquavita, Marco Bolis, Maddalena Fratelli, Cecile Rochette-Egly and Enrico Garattini

**SUPPLEMENTARY INFORMATION**

SUPPLEMENTARY METHODS pages 2-7

SUPPLEMENTARY REFERENCES pages 7-8

SUPPLEMENTARY TABLES 1-2 pages 9-10

SUPPLEMENTARY FIGURES 1-5 pages 11-18

SUPPLEMENTARY METHODS

*Reagents and anti-RAR antibodies*

Cycloheximide (CHX), ATRA and AM580 were from Calbiochem, Sigma and TOCRIS Bioscience. The following antibodies were used throughout the study: anti-RARβ, RPβ(F) ^1^; anti-RARα, RPα(F) ^2^; anti-RARγ, RPγ(F) ^3^; anti-RXRα, RPXα(A) {Bruck, 2005 #412}; anti-RARα, AB9α(F) and 10α(A1) ^2^; anti-RARα [RPalpha(F)] ^4^; anti-RARα [9α5A6] ^5^; anti-β-actin, anti-cEBPβ, anti-cEBPε, anti-STAT-1 and anti-N-CoR (Santa-Cruz-Biotechnology); anti-PU.1 (Cell Signaling); anti-paxillin (Transduction Laboratories); anti-ubiquitin mouse monoclonal antibody (Sigma). The anti-HSP27 (G3.1) and the anti-FABP5 (ab84028) antibodies were from ThermoFisher and Abcam.

*Stable Isotope Labeling by Amino Acids (SILAC), ATRA treatment, nuclear protein extraction and immunoprecipitation*

Stable isotope labeling by amino acids in cell culture (SILAC) was done on *MDA-MB453* cell lines expressing 3xFLAG-RARA or 3xFLAG-conjugated RARA-antisense. Cells were grown in SILAC DMEM (Thermo Scientific) with 10% dialyzed FBS (Thermo Scientific) supplemented with 50mg L-arginine and 50 mg L-Lysine (Thermo Scientific) for the light culture and with 50 mg L-arginine (U-13C6) and 50 mg L-lysine (U-13C6) for the heavy culture. Cells were maintained for six doublings in SILAC media to allow full amino acid incorporation. After six doublings, heavy labeled 3xFLAG-RARA or 3xFLAG-conjugated RARA-antisense cell lines were treated with 1 µM ATRA, the light counterparts were treated with vehicle (DMSO) for 1 hour. Cells were harvested and washed once in cold PBS solution. After centrifugation (250×g, 5 min, 4 °C), nuclear and nucleic acid binding proteins were extracted using the Qproteome Nuclear protein kit (Qiagen) according to the manufacturer's instructions. Equal amounts of each nuclear enriched fractions (NABP and INP, see Suppl. Fig. 1) obtained from the heavy and light cell samples were pre-cleared with A/G beads (Santa Cruz) for 3 hours at 4 °C. Thirty microliters of EZview Red Anti-FLAG M2 Affinity Gel (Sigma) was added to each nuclear fraction before overnight incubation at 4 °C to immunoprecipitate the bait protein. The beads were washed once with the dilution buffer before being combined into a new tube as shown in Suppl. Fig. 1. After sample combination, three more washes were performed. The beads were resuspended in SDS loading buffer for elution and incubated at 90 °C for 10 min. The eluate was separated on SDS/PAGE. An aliquot of each IP-nuclear extracts was submitted to Western blot analysis to confirm the presence of the RARA protein.

*Mass spectrometry analysis, protein identification and quantification*

Eluted nuclear fractions or whole cell proteome were separated by 1D 4-12% Nupage Novex Bis Tris Gel (Invitrogen), stained with Bio-Safe coomassie (Bio-Rad Laboratories) and digested with trypsin ^6^. Two µL of each sample were analysed on a Biobasic 18 column (150x0.18mm ID, particle size 5 µm, Thermo Scientific) coupled with an LTQ Orbitrap XL™ (Thermo Scientific). HPLC conditions were : column flow 2 µl/min; eluent A, H2O and 0.1% formic acid; eluent B, AcN and 0.1% formic acid; gradient program, from 2% of B to 60% of B in 240 min, then to 98% of B in 6 min for 4 min, and re-equilibration to 2% of B for 24 min. MS conditions were: source DESO Omni Spray (Prosolia, Indianapolis, IN) used in nanospray mode with positive ions; ion spray voltage, 2100 V; capillary temperature, 220 °C; capillary voltage, 42 V. MS spectra (m/z 400-2000) were acquired in the Orbitrap analyzer at 60,000 resolution, in parallel with the low-resolution MS/MS scans of the ten most abundant precursor ions being acquired in the LTQ, excluding singly charged ions. The lock-mass option was used to obtain the most accurate mass measurements in MS mode.

Mass spectra data processing was performed using Mascot Distiller (version 2.4.3.3, Matrix Science) with search and quantitation toolbox options. The generated de-isotoped peak list was submitted to an in-house Mascot server 2.3.02 for searching against the SwissProt database version 2016. Mascot search parameters were set as follows: species, Homo sapiens (20,160 sequences); enzyme, trypsin with maximal one missed cleavage; fixed modification: cysteine carbamidomethylation; variable modifications: methionine oxidation, label: 13C(6)15N(4) (R), and label:13C(6) (K);5 ppm mass tolerance for precursor peptide ions; 0.8 Da for MS/MS fragment ions; peptide charge: 2+, 3+. The default significance threshold (p<0.05) was set p< 0.01 in order to obtain for all searches a lower false discovery rate. The cut-off for peptide ion score was set to >30, a value ensuring an identification confidence > 99.9%. SILAC quantitation was performed in Mascot Distiller using SILAC K+6 R+10 quantitation method. To be accepted, peptides needed to pass the following quality thresholds: correlation threshold 0.95, fraction threshold 0.3 and standard error threshold 0.2. The median of the quantification values of all peptides belonging to the same protein determined the protein quantification value. For each experiment, the median value of heavy/light (H/L) ratio of quantified proteins were calculated. Quantified proteins with ≥ 1.5 and ≤ 1.5-H/L ratio were selected.

The mass spectrometry proteomics data have been deposited in the ProteomeXchange Consortium via the PRIDE partner repository with the dataset identifier PXD00876.

*Cell lines*

*NB4* and *HL-60* cells were obtained from the DSMZ repository (ACC 207), while *SK-BR-3* (HTB-30), *MCF-7* (HTB-22), *A549* (CRM-CCL-185) and *COS-7* (CRL-1651) cells were obtained from ATCC (American Type Culture Collection). *NB4* and *A549* cells were routinely passaged in RPMI1640 medium (10% FCS). *SK-BR-3* , *MCF-7* and *COS-7* cells were routinely cultured in DMEM/F12 medium (5% FCS).

*Silencing and over-expression of S100A3*

We generated *SK-BR-3, A549*, *NB4* and *HL-60* cell populations silenced for S100A3 infecting cells with lentiviral vectors (pGREENpuro, System Biosciences) containing the following shRNAs: *shSCRAM*, *shS100A3a*, *shS1003b* and *shS100A3c* or mixtures thereof. Custom-designed short hairpin RNAs (shRNAs) were synthesized by Ambion. To generate the corresponding lentiviral vectors, the following double-stranded oligonucleotides coding for S100A3 targeting shRNAs were inserted in the BamH1-EcoR1 sites of the pGREENpuro vector with the use of appropriate linker sequences:

*shS100A3a*, 5’-GGGAATGTGACTACAACAACTTCCTGTCAGATTGTTGTAGTCACATTCCC-3’

*shS100A3b*, 5’-GGTGGACTTTGTGGAGTATCTTCCTGTCAGAATACTCCACAAAGTCCACC-3’

*shS100A3c*, 5’-ACGAGTACTTCAAGGACTGCTTCCTGTCAGACAGTCCTTGAAGTACTCGT-3’

The following scrambled shRNA construct derived from the sequence of *shS100A3a* was used as a negative control:

*shSCRAM*, 5’-GACGAAGACTAACATAGGTCTTCCTGTCAGAACCTATGTTAGTCTTCGTC-3’

For these experiments, we used standard infection protocols (System Biosciences). Following infection, *SK-BR-3* were selected in puromycin (1.0 μg/ml) containing F12 medium (5% bovine serum), while *A549* and *NB4* cells were selected in puromycin (1.0 μg/ml) containing RPMI medium (10% bovine serum), for at least 15 days. Subsequent passages of the cell populations were performed in complete F12 or RPMI medium containing puromycin (0.5 μg/ml).

*Immunofluorescence microscopy*

Cells were fixed with 3% paraformaldehyde and permeabilized with 0.1% Triton X 100 in PBS. Fixed cells were incubated with the anti-S100A3 mouse monoclonal antibody (sc-514339, Santa Cruz) and the anti-RARα rabbit polyclonal antibody (sc-551, Santa Cruz). Positive cells were identified following further incubation for with 488 or 543 Alexa-conjugated secondary antibodies (Thermo Scientific). Hoechst was used for nuclear detection. Images were recorded using a confocal laser microscopy (IX81 microscope coupled to Olympus FluorView 300 laser scanning confocal microscope, Olympus).

*Immunoprecipitation, Far-western and GST pull-down assays in COS-7 cells*

*COS-7* cells were transfected with the pcDNA3 plasmid containing the S100A3 cDNA. Extracts were precipitated with a S100A3 monoclonal antibody (sc-514339, Santa Cruz Biotechnology). The immuno-precipitates were subjected to Far-Western analysis using the following GST-tagged recombinant proteins corresponding to RARα and derived deletion mutants: *GST-RARα*, *GST-ABC*, *GST-DEF* and *GST-DEFΔH12* ^7^. For the pull-down experiments, we generated a GST-S100A3 construct in the *pGEX4T1* plasmid allowing expression of the recombinant tagged protein in the *BL21* E. coli strain. To this purpose the S100A3 coding region was amplified by RT-PCR from *SK-BR-3* cells and inserted into the EcoRI-XhoI site of the plasmid. The following S100A3 amplimers containing a 5’ EcoR1 site and a 5’ XhoI site (italics), respectively, were used:

sense, 5’-*TAGAATTCGG* ATGGCCAGGCCTCTGGAG-3’ (nt 84-101, NM_002960.1)

anti-sense, 5’-*TACTC* GAGGCTACTGGGAGCAGGGG-3’ (compl. to nt 374-393, NM_002960.1).

The transfection experiments involving N-CoR were conducted with a pSG5 plasmid allowing the expression of a cDNA encoding the N-CoR fragment (aa. 1629-2453) containing the RARα-binding domains (C. Rochette-Egly, unpublished results).

The recombinant S100A3 protein conjugated to Glutathione-sepharose beads (Amersham) was incubated with extracts of *COS-7* cells transfected with the pSG5 plasmids containing RARα, PML-RARα, RARα2, RARβ, RARγ, PML or the RARα point as well as deletion mutants described in Suppl. Fig.4 for 4 hours. Pulled-down proteins were subjected to WB analysis using anti-RARα or anti-GST antibodies.

*Densitometric analyses of the Western blots*

Densitometric analysis was performed with the Progenesis software (Nonlinear Dynamics Co.) using routine protocols ^8^.

SUPPLEMENTARY REFERENCES

1 Rochette-Egly C, Gaub MP, Lutz Y, Ali S, Scheuer I, Chambon P. Retinoic acid receptor-beta: immunodetection and phosphorylation on tyrosine residues. Mol Endocrinol 1992; 6: 2197-2209.

2 Gianni M, Fratelli M, Bolis M, Kurosaki M, Zanetti A, Paroni G et al. RARalpha2 and PML-RAR similarities in the control of basal and retinoic acid induced myeloid maturation of acute myeloid leukemia cells. Oncotarget 2016.

3 Bastien J, Adam-Stitah S, Riedl T, Egly JM, Chambon P, Rochette-Egly C. TFIIH interacts with the retinoic acid receptor gamma and phosphorylates its AF-1-activating domain through cdk7. J Biol Chem 2000; 275: 21896-21904.

4 Bruck N, Bastien J, Bour G, Tarrade A, Plassat JL, Bauer A et al. Phosphorylation of the retinoid x receptor at the omega loop, modulates the expression of retinoic-acid-target genes with a promoter context specificity. Cell Signal 2005; 17: 1229-1239.

5 Gaub MP, Rochette-Egly C, Lutz Y, Ali S, Matthes H, Scheuer I et al. Immunodetection of multiple species of retinoic acid receptor alpha: evidence for phosphorylation. Exp Cell Res 1992; 201: 335-346.

6 Brunelli L, Llansola M, Felipo V, Campagna R, Airoldi L, De Paola M et al. Insight into the neuroproteomics effects of the food-contaminant non-dioxin like polychlorinated biphenyls. J Proteomics 2012; 75: 2417-2430.

7 Gianni M, Fratelli M, Bolis M, Kurosaki M, Zanetti A, Paroni G et al. RARalpha2 and PML-RAR similarities in the control of basal and retinoic acid induced myeloid maturation of acute myeloid leukemia cells. Oncotarget 2017; 8: 37041-37060.

8 Pasetto L, Pozzi S, Castelnovo M, Basso M, Estevez AG, Fumagalli S, De Simoni MG, Castellaneta V, Bigini P, Restelli E, Chiesa R, Trojsi F, Monsurrò MR, Callea L, Malešević M, Fischer G, Freschi M, Tortarolo M, Bendotti C, Bonetto V. Targeting Extracellular Cyclophilin A Reduces Neuroinflammation and Extends Survival in a Mouse Model of Amyotrophic Lateral Sclerosis. J Neuroscience 2017; 37: 1413-1427.

| **Protein Accession** | **Protein Description** | **Mass (kDa)** | **H/L**  **ratio** | **unique peptides** |
| --- | --- | --- | --- | --- |
| ***NABP fraction*** |  |  |  |  |
| ROA0_HUMAN | Heterogeneous nuclear ribonucleoprotein A0 | 31.0 | 1.09 | 1 |
| H2A1A_HUMAN | Histone H2A type 1-A | 14.2 | 1.55 | 2 |
| ***INP fraction*** |  |  |  |  |
| CEP83_HUMAN | Centrosomal protein of 83 kDa | 82.2 | 1.80 | 2 |
| H2AW_HUMAN | Core histone macro-H2A.2 | 40.7 | 2.03 | 1 |
| H10_HUMAN | Histone H1.0 | 20.9 | 1.60 | 6 |
| H2A1C_HUMAN | Histone H2A type 1-C | 14.1 | 1.74 | 7 |
| H2A2B_HUMAN | Histone H2A type 2-B | 14.2 | 1.77 | 4 |
| H2AV_HUMAN | Histone H2A.V | 13.5 | 1.79 | 9 |
| H2B1C_HUMAN | Histone H2B type 1-C | 13.9 | 1.85 | 10 |
| H31T_HUMAN | Histone H3.1t | 15.6 | 1.81 | 20 |
| RL1D1_HUMAN | Ribosomal L1 domain-containing protein 1 | 55.9 | 1.75 | 1 |

**Supplementary Table 1** *List of the nuclear RARα-interacting proteins whose interaction is stimulated by ATRA in RA-453 cells* The table lists the nuclear RARα-interacting proteins whose interaction is stimulated by ATRA in *RA-453* cells. The table shows the nuclear proteins belonging to the *NABP* (*Nucleic Acid Binding Proteins*) and the *INP* (*Insoluble Nuclear Proteins*) fractions which were shown to interact specifically with unliganded RARα and whose interaction is stimulated following challenge of *RA-453* cells with ATRA (1 µM) for 1 hour. The ATRA-dependent increase in the RARα binding of each protein is shown in column 4 where the heavy/light isotope ratio (*H/L*) is indicated. The number of unique peptides identified for each protein by mass-spectrometry is shown in the last column.

| **Protein Accession** | **Protein Description** | **Mass (kDa)** | **H/L ratio** | **unique peptides** |
| --- | --- | --- | --- | --- |
| ***NABP fraction*** |  |  |  |  |
| GTF2I_HUMAN | General transcription factor II-I | 112.9 | 0.85 | 2 |
| HNRPK_HUMAN | Heterogeneous nuclear ribonucleoprotein K | 51.2 | 0.71 | 1 |
| KHDR1_HUMAN | KH domain-containing, RNA-binding, signal transduction-associated protein 1 | 48.3 | 0.71 | 1 |
| LRC15_HUMAN | Leucine-rich repeat-containing protein 15 | 65.2 | 0.01 | 2 |
| PSPC1_HUMAN | Paraspeckle component 1 | 59.5 | 0.74 | 1 |
| RBM3_HUMAN | Putative RNA-binding protein 3 | 17.2 | 0.87 | 1 |
| RU17_HUMAN | U1 small nuclear ribonucleoprotein 70 kDa | 51.6 | 0.76 | 1 |
|  |  |  |  |  |
| ***INP fraction*** |  |  |  |  |
| DSG4_HUMAN | Desmoglein-4 | 115.5 | 0.01 | 1 |
| FABP5_HUMAN | Fatty acid-binding protein 5 | 15.5 | 0.01 | 2 |
| HSP7C_HUMAN | Heat shock cognate 71 kDa protein | 71.1 | 0.01 | 1 |
| HSPB1_HUMAN | Heat shock protein beta-1 (HSPB1 or HSP27) | 22.8 | 0.01 | 1 |
| ROA0_HUMAN | Heterogeneous nuclear ribonucleoprotein A0 | 31.0 | 0.72 | 1 |
| AT8B2_HUMAN | Phospholipid-transporting ATPase | 138.6 | 0.01 | 1 |
| PKP3_HUMAN | Plakophilin-3 | 87.5 | 0.78 | 2 |
| S10A3_HUMAN | Protein S100-A3 | 12.3 | 0.01 | 1 |
| SBP1_HUMAN | Selenium-binding protein 1 | 52.9 | 0.01 | 3 |
| RS27A_HUMAN | Ubiquitin-40S ribosomal protein S27a | 18.3 | 0.01 | 2 |

**Supplementary Table 2** *List of the nuclear RARα-interacting proteins whose interaction is reduced by ATRA in RA-453 cells* The table lists the nuclear RARα-interacting proteins whose interaction is reduced by ATRA in *RA-453* cells. The table illustrates the nuclear proteins belonging to the *NABP* (*Nucleic Acid Binding Proteins*) and the *INP* (*Insoluble Nuclear Proteins*) fractions which were shown to interact specifically with unliganded RARα and whose interaction is stimulated following challenge of *RA-453* cells with ATRA (1 µM) for 1 hour. The ATRA-dependent decrease in the RARα binding of each protein is shown in column 4 where the heavy/light isotope ratio (*H/L*) is indicated. The number of unique peptides identified for each protein by mass-spectrometry is shown in the last column. The interacting proteins which were the object of further studies are marked in red.

**
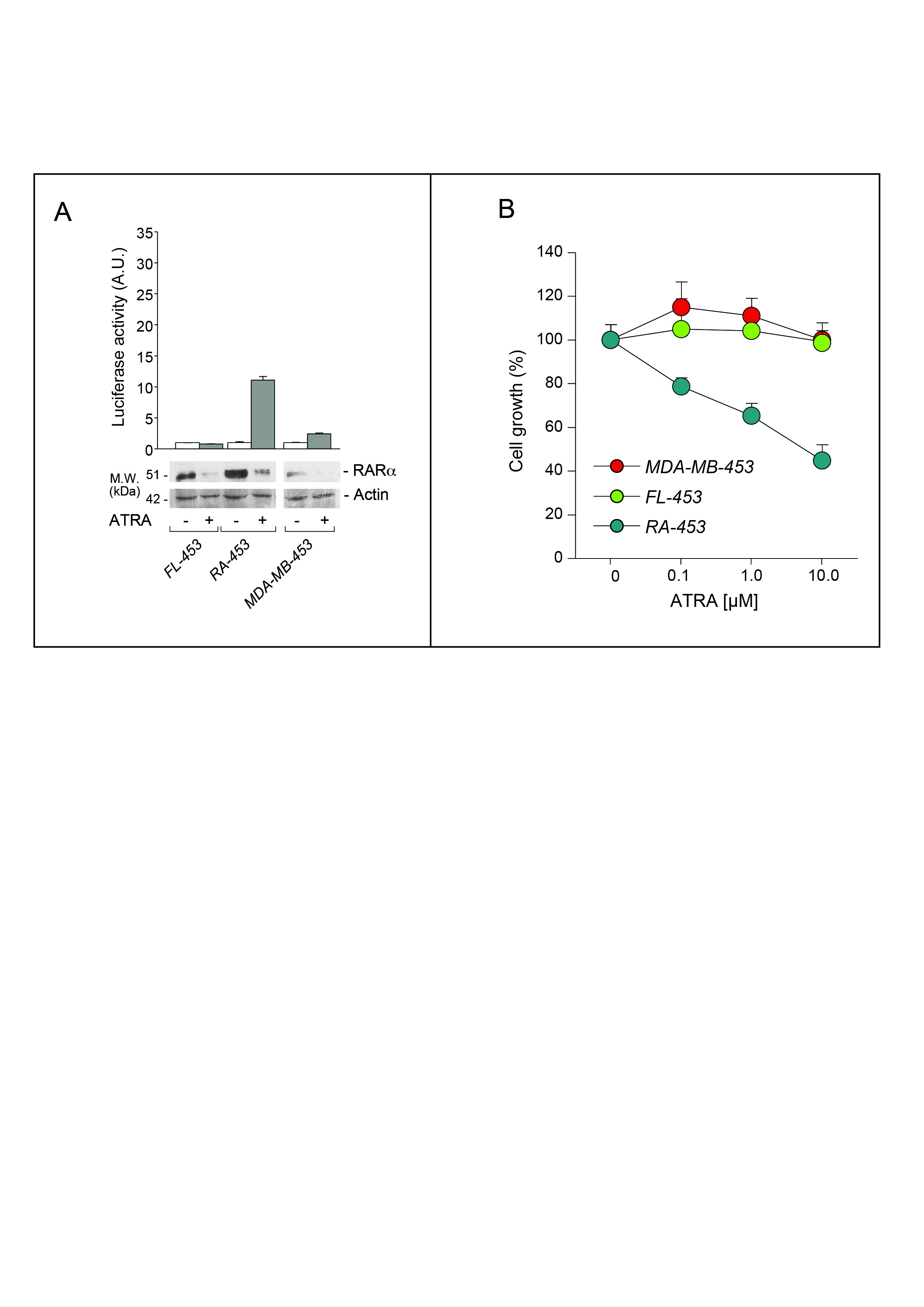
**

**Suppl. Fig. 1** *Co-immunoprecipitation immunofluorescence studies on the interaction between RARα and S100A3*

We generated two cell lines (*RA-453* and *FL-453*) stably transfected with an expression plasmid containing an N-terminally FLAG-tagged version of human RARα (*RA-453*) and the corresponding FLAG-only control (*FL-453*) from the ATRA-insensitive HER2^+^/ER^-^ *MDA-MB-453* cell line. (**A**) The Western blots illustrate the levels of RARα and actin in *RA-453*, *FL-453* and *MDA-MB-453* cell lines grown in the presence of vehicle (DMSO) or ATRA (1.0 µM) for 24 hours. The upper bar-graphs show the levels of luciferase activity observed in the same cell lines following transient transfection of the retinoid-dependent luciferase reporter construct (*β2RARE-Luc*) and treatment with (DMSO) or ATRA (1.0 µM) for 24 hours. Each value is the Mean+SD of 3 replicate cultures. (**B**) The panel illustrates the growth of *RA-453*, *FL-453* and *MDA-MB-453* cells following treatment with the indicated concentrations of ATRA for 96 hours.

**
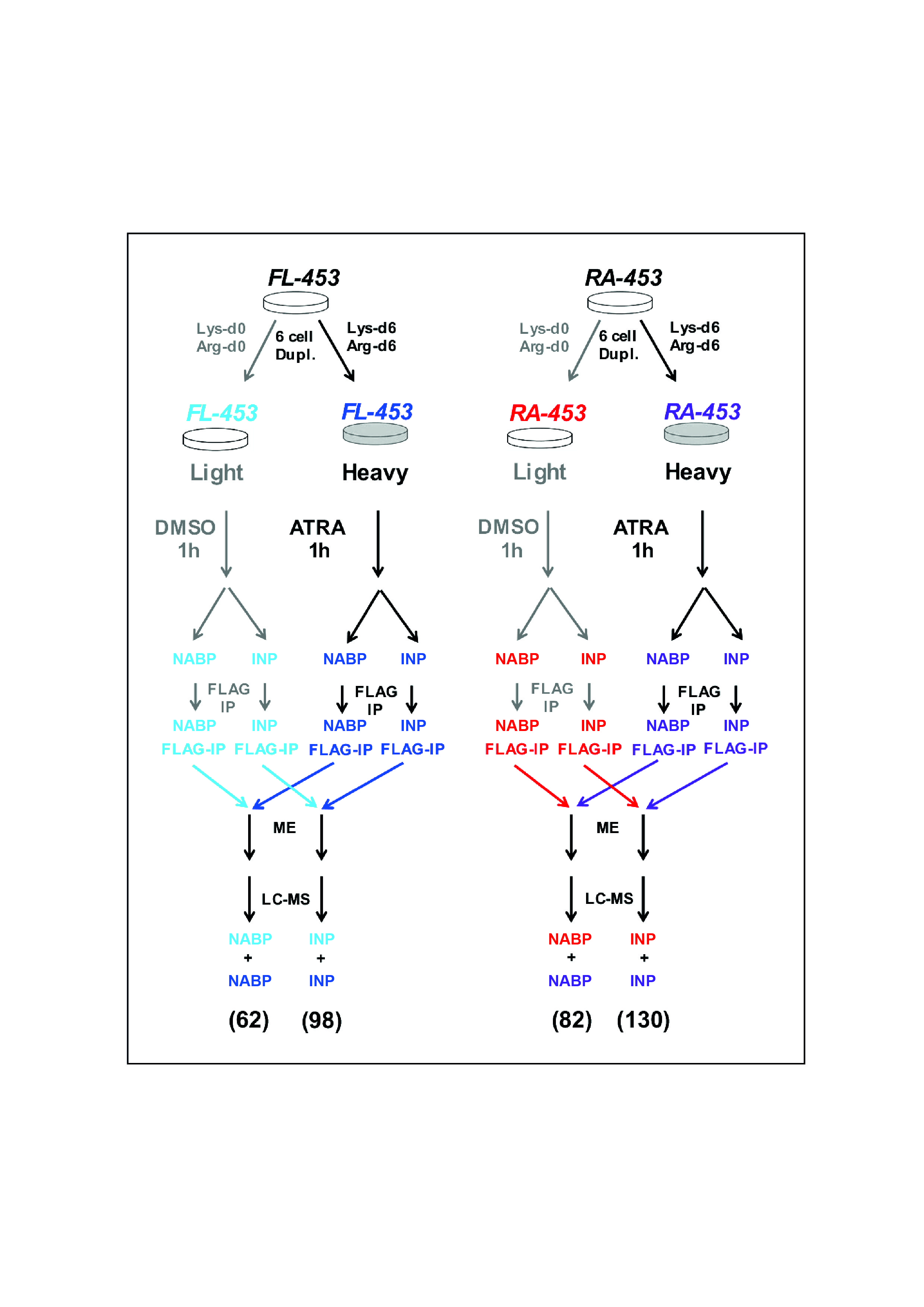
**

**Suppl. Fig. 2** *Identification of* *RARα interacting proteins: flow chart of the experimental design*

The *RA-453* and *FL-453* couple of cells were incubated in the presence of the indicated deuterium labeled Lys and Arg amino acids (Lys-d6 and Arg-d6, Heavy) or regular amino acids (Lys-d0 and Arg-d0, Light). Subsequently, cells were treated with ATRA (1µM) or vehicle (DMSO). At the end of the treatment the indicated nuclear extracts were subjected to RARα interactomic analysis involving the steps illustrated in the flow chart. NABP = *Nucleic Acid Binding Proteins* fraction; INP = *Insoluble Nuclear Proteins* fraction; ME = Monodimensional Electrophoresis; LC-MS = liquid chromatography tandem mass spectrometry; Dupl. = Duplications; FLAG-IP = Immunoprecipitation with the use of anti-FLAG antibodies.

**A**

**B**

**C**


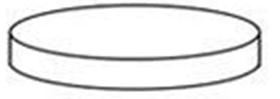

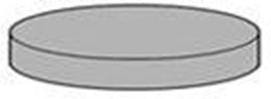


**FL453**

**FL453**

**Light**

**Heavy**

**Lys-d0**

**Arg-d0**

**Lys-d6**

**Arg-d6**

**ANE**

**HNE**

**DMSO**

**1h**

**ANE**

**HNE**

**ATRA**

**1h**

**FLAG**

**IP**

**ANE**

**FLAG-IP**

**HNE**

**FLAG-IP**

**ME**

**LC-MS**

**ANE**

**+**

**ANE**

**Proteins**

**(62)**

**-**

**-**

**Acidic RARα**

**interacting proteins**

**FL453**

**6 cell**

**Dupl.**


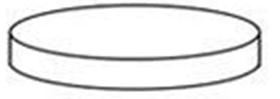


**FLAG**

**IP**

**ANE**

**FLAG-IP**

**HNE**

**FLAG-IP**

**HNE**

**+**

**HNE**

**Proteins**

**(98)**


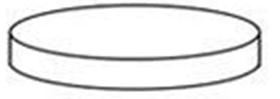

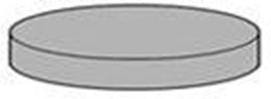


**RA453**

**RA453**

**Light**

**Heavy**

**Lys-d0**

**Arg-d0**

**Lys-d6**

**Arg-d6**

**ANE**

**HNE**

**DMSO**

**1h**

**ANE**

**HNE**

**ATRA**

**1h**

**FLAG**

**IP**

**ANE**

**FLAG-IP**

**HNE**

**FLAG-IP**

**ME**

**LC-MS**

**ANE**

**+**

**ANE**

**Proteins**

**(82)**

**Histonic RARα**

**interacting proteins**

**RA453**

**6 cell**

**Dupl.**


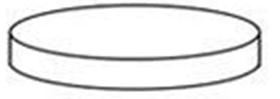


**FLAG**

**IP**

**ANE**

**FLAG-IP**

**HNE**

**FLAG-IP**

**HNE**

**+**

**HNE**

**Proteins**

**(130)**

**
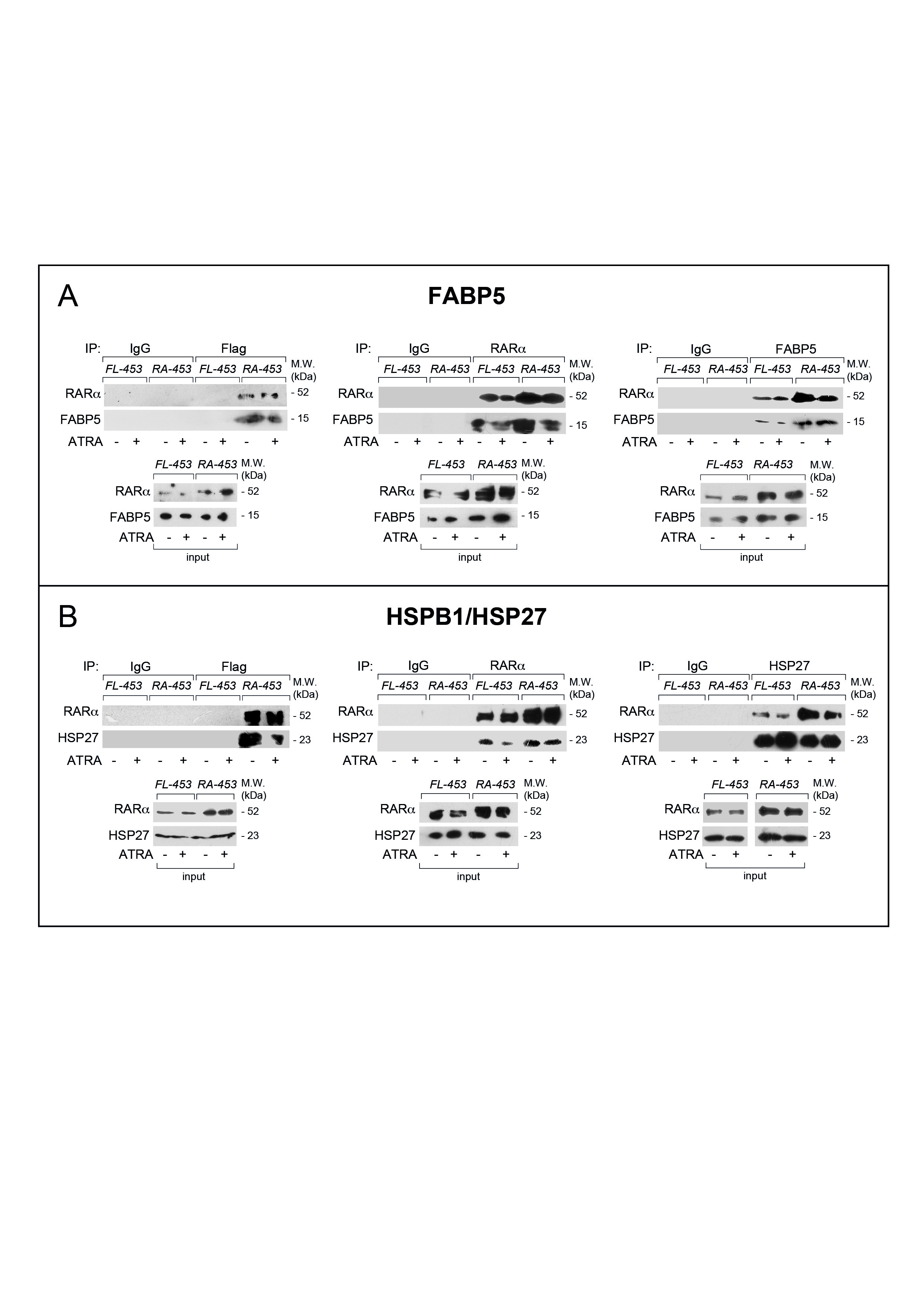
**

**Suppl. Fig. 3** *Co-immunoprecipitation studies on the interaction between RAR, FABP5 and HSPB1/HSP27*

*FL-453* and *RA-453* cells were treated with vehicle (DMSO) or ATRA (1 µM) for 1 hour. At the end of the treatment, total cell extracts were immunoprecipitated with: (**A**) anti-FLAG mouse monoclonal antibodies (left), anti-RARα mouse monoclonal antibodies (middle) and anti-FABP5 mouse monoclonal antibodies (right) or the corresponding non-specific immunoglobulins G (IgG) as negative controls. (**B**) the same anti-FLAG, anti-RARα antibodies and control IgGs as in (**A**) as well as anti-HSP27 mouse monoclonal antibodies (right) were used. The various immunoprecipitates were subjected to Western blot analysis with an anti-RARα rabbit polyclonal antibody or the same anti-FABP5 (**A**) and anti-HSP27 (**B**) antibodies, as indicated. Input = Western blot analysis of the cell extracts before the indicated immunoprecipitation step. Each immunoprecipitation is representative of at least two independent experiments providing the same type of results.

**
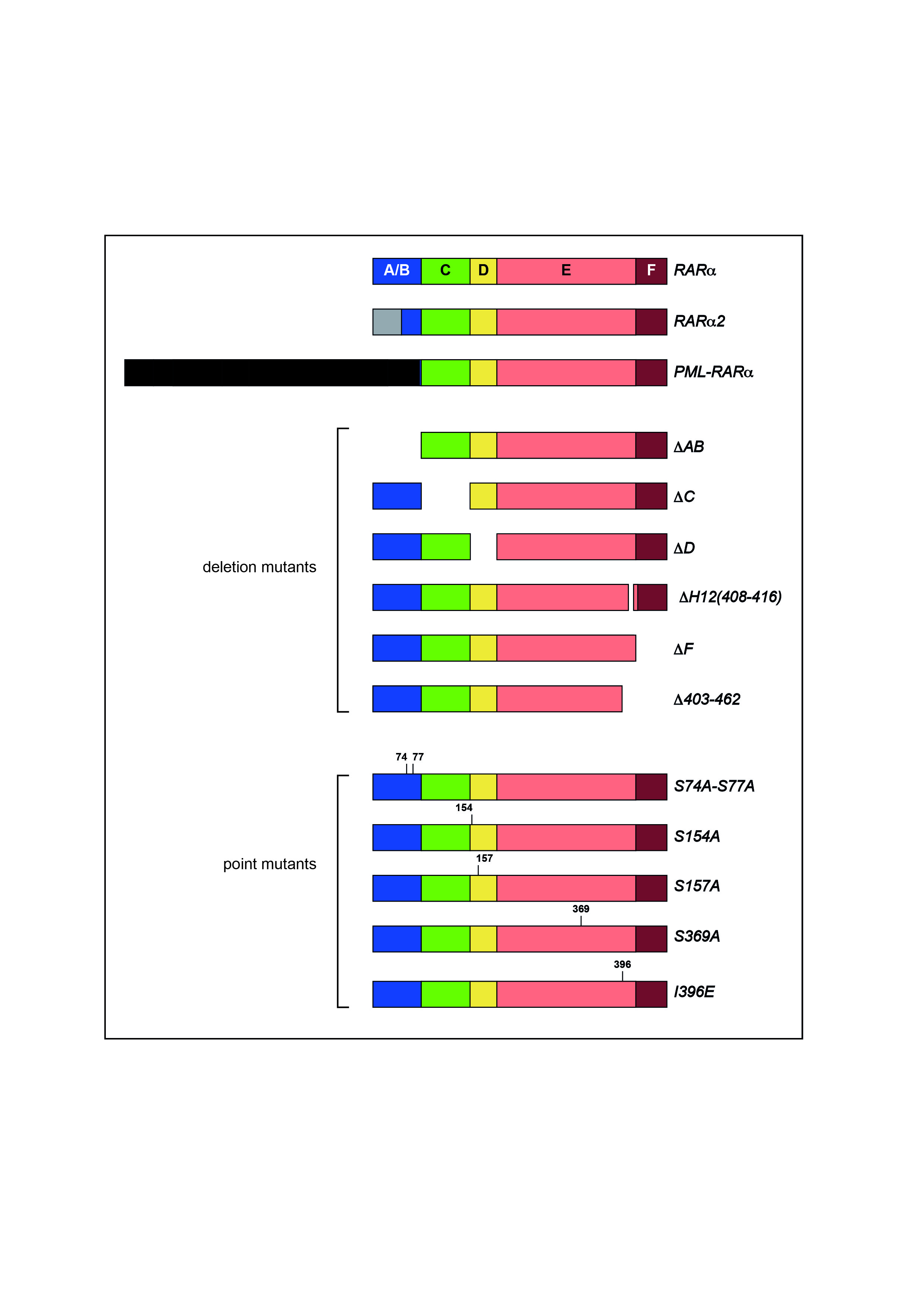
**

**Suppl. Fig. 4** *Structure of the RARa mutants used in the GST pull-down experiments*

The figure illustrates the general structure of RARα, RARα2, PML-RARα and the indicated deletion and point mutants. The different regions of RARα are indicated with a different color. From the amino-terminal to the carboxy-terminal end: A/B = ligand-independent transactivation regions (blue); C = DNA-binding region (green); D = hinge region (yellow); ligand-binding region (pink); F = c-terminal region (brown).

**A**

**B**

**C**


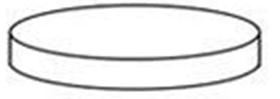

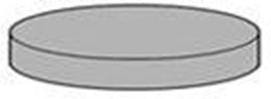


**FL453**

**FL453**

**Light**

**Heavy**

**Lys-d0**

**Arg-d0**

**Lys-d6**

**Arg-d6**

**ANE**

**HNE**

**DMSO**

**1h**

**ANE**

**HNE**

**ATRA**

**1h**

**FLAG**

**IP**

**ANE**

**FLAG-IP**

**HNE**

**FLAG-IP**

**ME**

**LC-MS**

**ANE**

**+**

**ANE**

**Proteins**

**(62)**

**-**

**-**

**Acidic RARα**

**interacting proteins**

**FL453**

**6 cell**

**Dupl.**


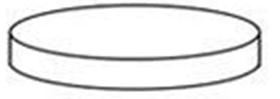


**FLAG**

**IP**

**ANE**

**FLAG-IP**

**HNE**

**FLAG-IP**

**HNE**

**+**

**HNE**

**Proteins**

**(98)**


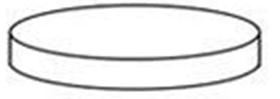

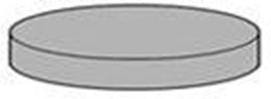


**RA453**

**RA453**

**Light**

**Heavy**

**Lys-d0**

**Arg-d0**

**Lys-d6**

**Arg-d6**

**ANE**

**HNE**

**DMSO**

**1h**

**ANE**

**HNE**

**ATRA**

**1h**

**FLAG**

**IP**

**ANE**

**FLAG-IP**

**HNE**

**FLAG-IP**

**ME**

**LC-MS**

**ANE**

**+**

**ANE**

**Proteins**

**(82)**

**Histonic RARα**

**interacting proteins**

**RA453**

**6 cell**

**Dupl.**


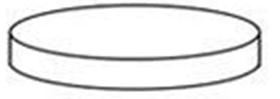


**FLAG**

**IP**

**ANE**

**FLAG-IP**

**HNE**

**FLAG-IP**

**HNE**

**+**

**HNE**

**Proteins**

**(130)**

**
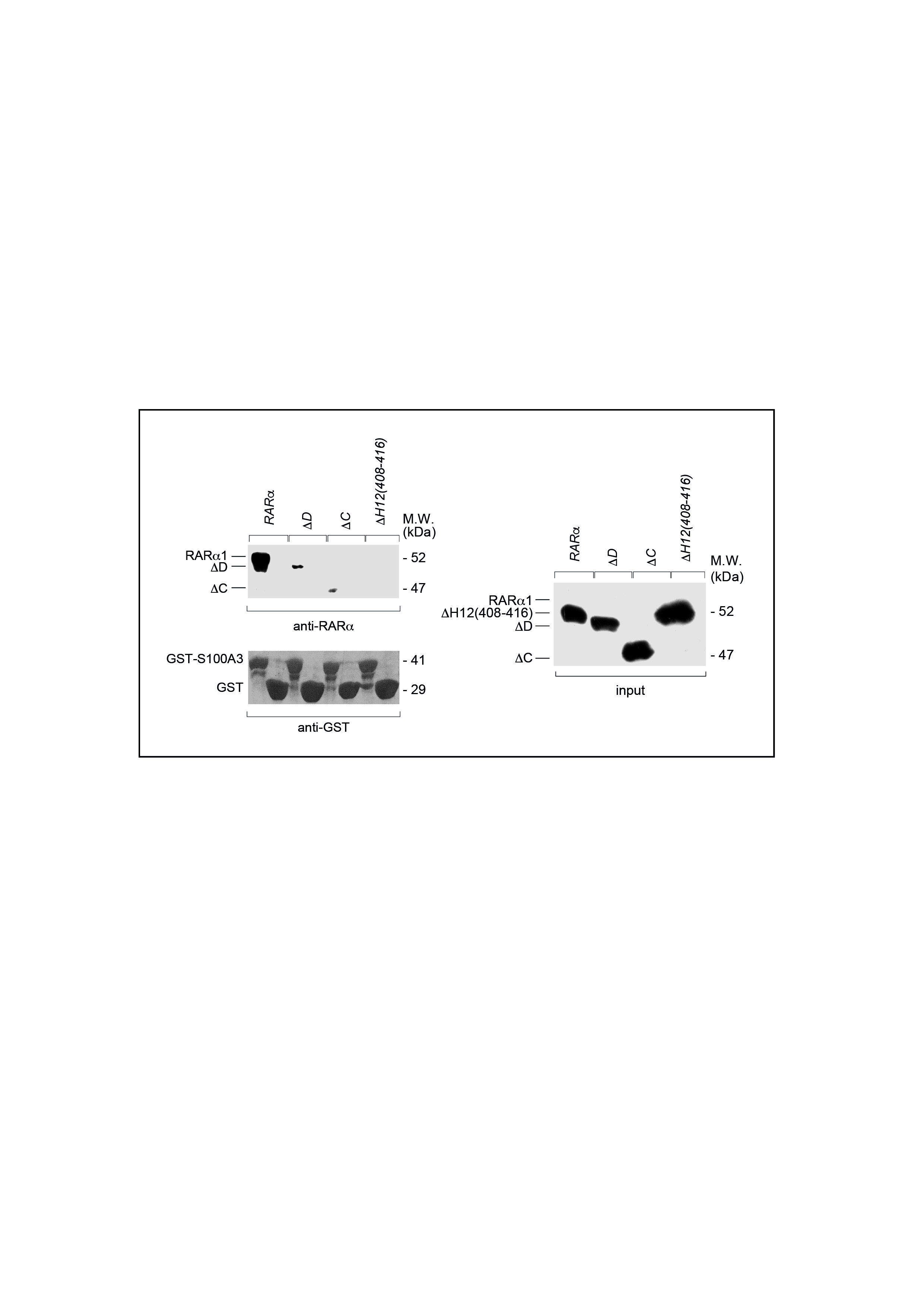
**

**Suppl. Fig. 5** *Pull-down experiments with the ΔC* *and* *ΔD mutants of RARa*

*COS-7* cells were co-transfected with equal amounts of RARα, the *ΔC*, *ΔD* and *ΔH12(408-416)* deletion mutants, as indicated. The GST-tagged recombinant protein, GST-S100A3, and the GST negative control were used for the pull-down experiments. The two recombinant proteins conjugated to Glutathione-Sepharose beads were incubated with extracts of transfected *COS-7* cells. GST pull-down precipitates were blotted on nitro-cellulose filters, hybridized with anti-RARα antibodies. Subsequently the filters were re-blotted with an anti-GST antibody, as indicated. Input: cell extracts (15 μg of protein) representing 10% of the total amount of protein were subjected to Western blot analysis with the above anti-RARα antibody.


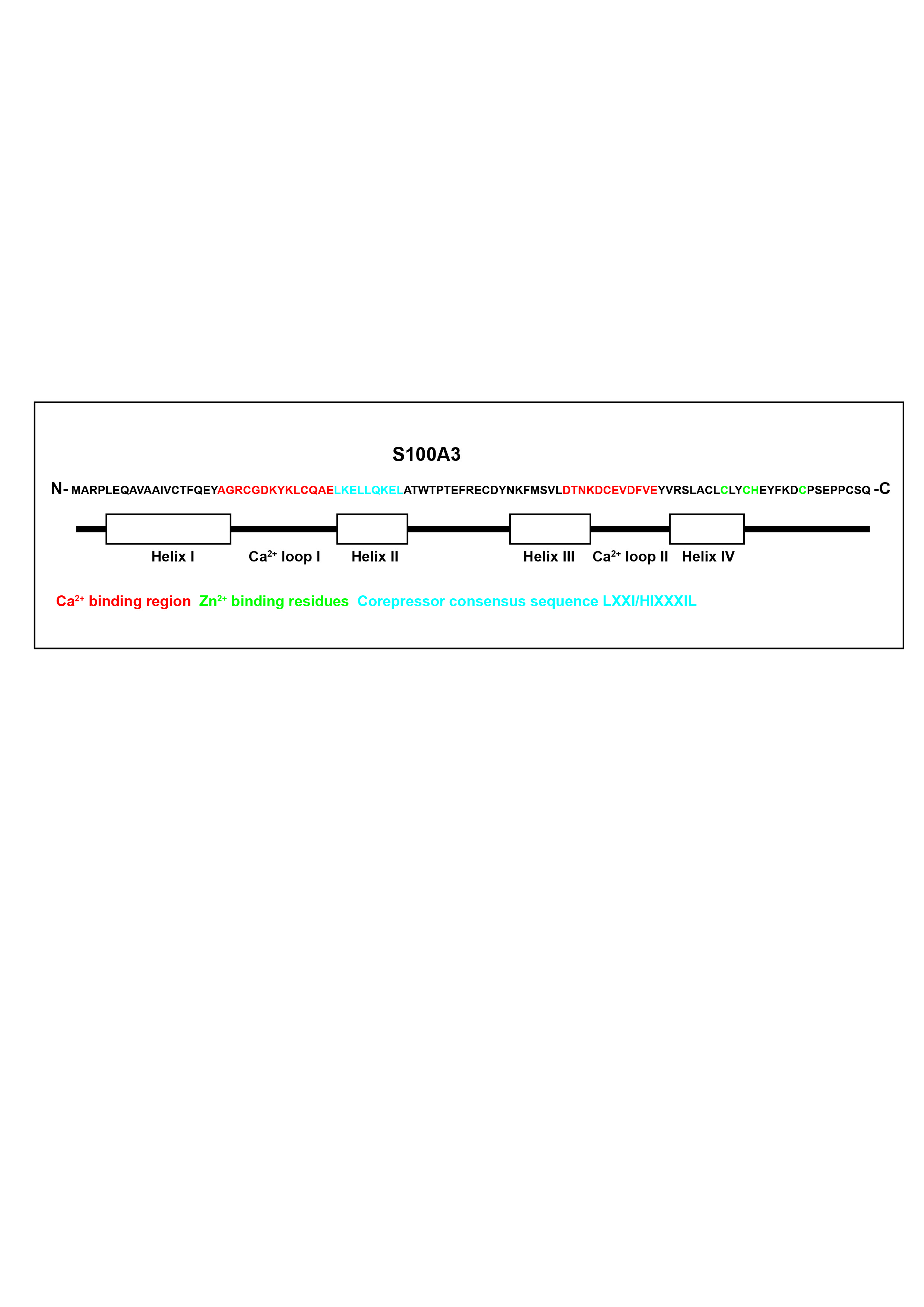


**Suppl. Fig. 6** *Sequence and schematic structure of the S100A3 protein*

The panel illustrates the amino acid sequence and the primary structural elements of the S100A3 protein as indicated.

**
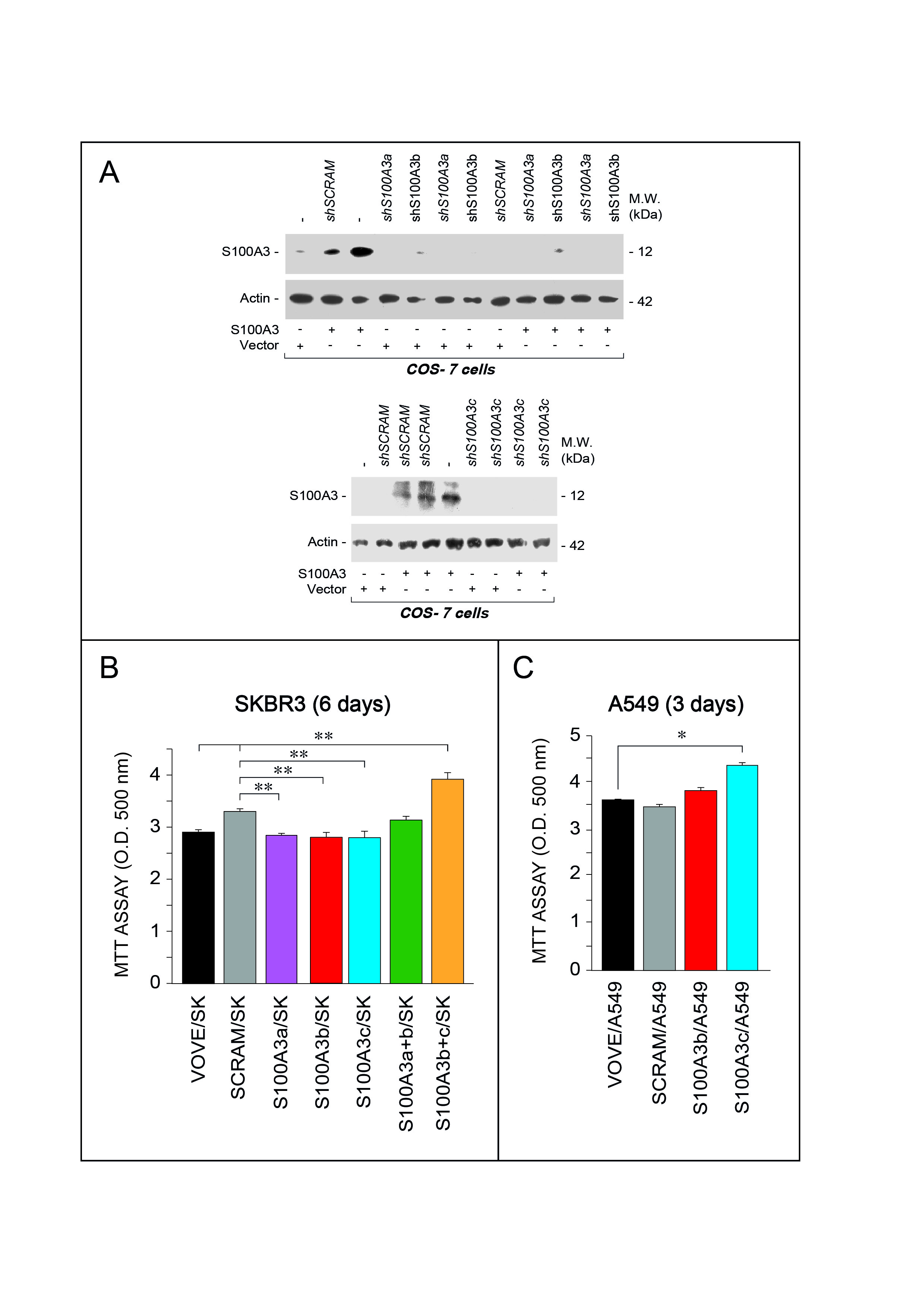
**

**Suppl. Fig. 7** *Specificity of the shRNAs targeting S100A3 and functional studies on the basal growth of SK-BR-3 and A549 cells*

(**A**) To define the specificity of the three S100A3 shRNA lentiviral constructs used for the functional studies, *COS-7* cells were transiently co-transfected with an expression plasmid for the S100A3 cDNA (*S100A3*) or the corresponding void vector (-) and the indicated S100A3 targeting shRNAs (*shS100A3a*; *shS100A3b* and *shS100A3c*) or scramble shRNA (*shSCRAM*). Twenty four hours following transfection, cell extracts were subjected to Western blot analysis with anti-S100A3 and anti-actin antibodies. (**B**) *SK-BR-3* cell populations stably infected with the void lentiviral vector (*VOVE/SK*), the scrambled negative control shRNA (*SCRAM/SK*), the indicated S100A3 targeting shRNA lentiviral constructs and combinations thereof were grown for 6 days in standard conditions. The growth of each cell population was evaluated with the use of the MTT assay. Each experimental point is the mean+S.E. of 4 independent cell cultures. **Significantly different (p<0.01 according to a two-way Student’s t-test). (**C**) *VOVE/A549*, *SCRAM/A549*, *S100A3b/A549* and *S100A3c/A549* cells were grown for 3 days in standard conditions. The growth of each cell populations was evaluated with the use of the MTT assay. Each experimental point is the Mean+S.E. of 4 independent cell cultures. **Significantly different (** p<0.01 according to a two-way Student’s t-test).

**
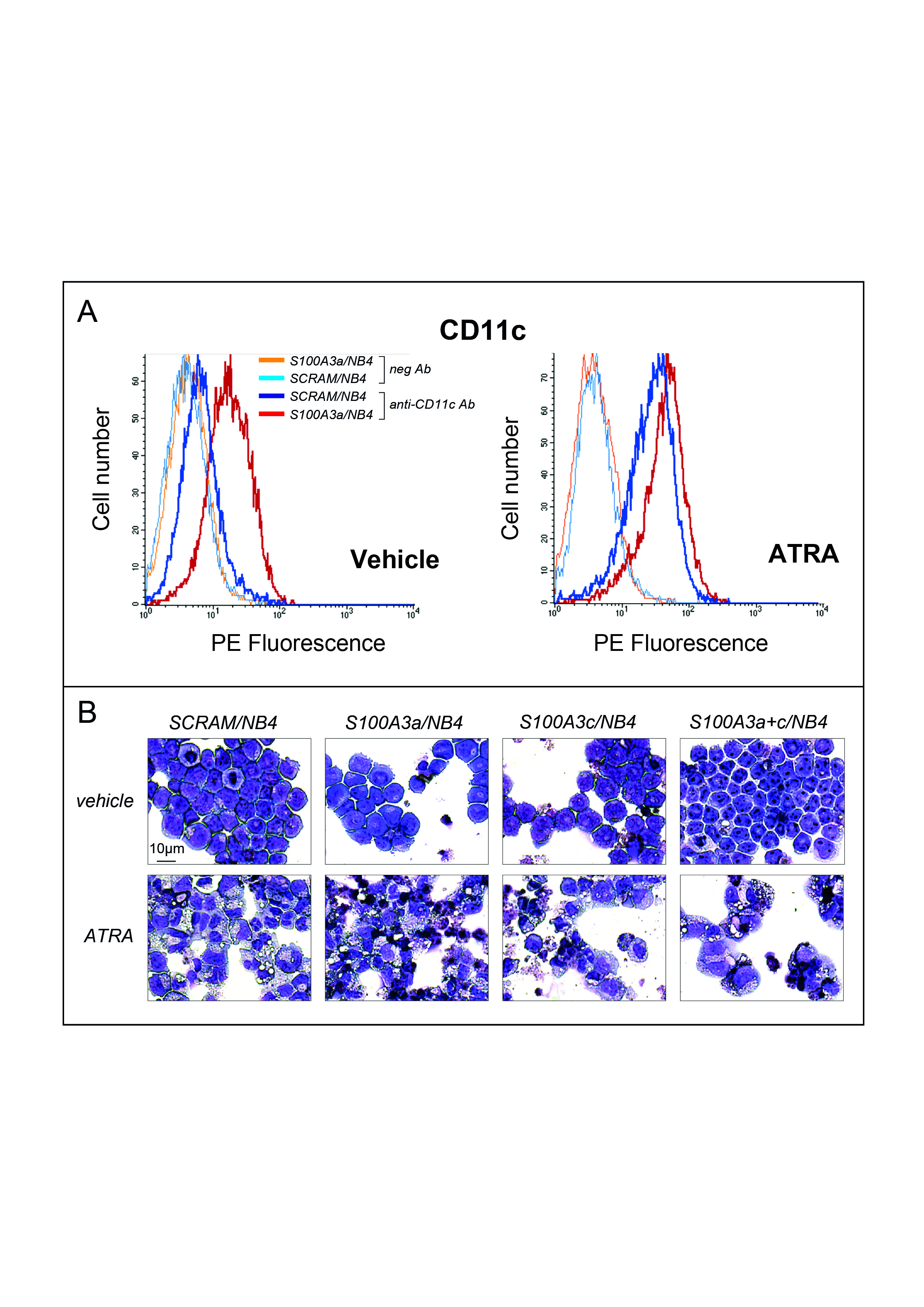
**

**Suppl. Fig. 8** *Specificity of the shRNAs targeting S100A3 and functional studies in NB4 cells*

(**A**) CD11c expression in S100A3 shRNA expressing NB4 cells: the panel illustrates representative FACS analyses of *SCRAM/NB4* and *S100A3a/NB4* treated with vehicle or ATRA (10^-8^ M) for 2 days. Cells were labeled with an anti-CD11c antibodies (*anti-CD11c Ab*) and negative-control IgG (*neg Ab*), as indicated. PE = phycoerythrin. (**B**) Morphology of S100A3 shRNA expressing NB4 cells: NB4 cell populations stably infected with lentiviral vectors expressing the indicated S100A3 targeting shRNAs were treated with vehicle or ATRA (10^-7^ M) for 3 days. Cell cytospins on microscopy glass slides were fixed and stained with the Diff Stain Quick kit (Pro-Eko, srl).
